# BMP2-dependent gene regulatory network analysis reveals *Klf4* as a novel transcription factor of osteoblast differentiation

**DOI:** 10.1038/s41419-021-03480-7

**Published:** 2021-02-19

**Authors:** Shuaitong Yu, Jinqiang Guo, Zheyi Sun, Chujiao Lin, Huangheng Tao, Qian Zhang, Yu Cui, Huanyan Zuo, Yuxiu Lin, Shuo Chen, Huan Liu, Zhi Chen

**Affiliations:** 1 State Key Laboratory Breeding Base of Basic Science of Stomatology (Hubei-MOST) and Key Laboratory for Oral Biomedicine of Ministry of Education (KLOBM), School and Hospital of Stomatology, Wuhan University, Wuhan, China; 2 Department of Developmental Dentistry, University of Texas Health Science Center, San Antonio, TX USA; 3 Department of Periodontology, School and Hospital of Stomatology, Wuhan University, Wuhan, China

**Keywords:** Bone development, Differentiation

## Abstract

Transcription factors (TFs) regulate the expression of target genes, inducing changes in cell morphology or activities needed for cell fate determination and differentiation. The BMP signaling pathway is widely regarded as one of the most important pathways in vertebrate skeletal biology, of which BMP2 is a potent inducer, governing the osteoblast differentiation of bone marrow stromal cells (BMSCs). However, the mechanism by which BMP2 initiates its downstream transcription factor cascade and determines the direction of differentiation remains largely unknown. In this study, we used RNA-seq, ATAC-seq, and animal models to characterize the BMP2-dependent gene regulatory network governing osteoblast lineage commitment. *Sp7-*Cre*; Bmp2*^fx/fx^ mice (BMP2-cKO) were generated and exhibited decreased bone density and lower osteoblast number (*n* > 6). In vitro experiments showed that BMP2-cKO mouse bone marrow stromal cells (mBMSCs) had an impact on osteoblast differentiation and deficient cell proliferation. Osteogenic medium induced mBMSCs from BMP2-cKO mice and control were subjected to RNA-seq and ATAC-seq analysis to reveal differentially expressed TFs, along with their target open chromatin regions. Combined with H3K27Ac CUT&Tag during osteoblast differentiation, we identified 2338 BMP2-dependent osteoblast-specific active enhancers. Motif enrichment assay revealed that over 80% of these elements were directly targeted by RUNX2, DLX5, MEF2C, OASIS, and KLF4. We deactivated *Klf4* in the *Sp7* + lineage to validate the role of KLF4 in osteoblast differentiation of mBMSCs. Compared to the wild-type, *Sp7-*Cre*; Klf4*^fx/+^ mice (KLF4-Het) were smaller in size and had abnormal incisors resembling BMP2-cKO mice. Additionally, KLF4-Het mice had fewer osteoblasts and decreased osteogenic ability. RNA-seq and ATAC-seq revealed that KLF4 mainly “co-bound” with RUNX2 to regulate downstream genes. Given the significant overlap between KLF4- and BMP2-dependent NFRs and enriched motifs, our findings outline a comprehensive BMP2-dependent gene regulatory network specifically governing osteoblast differentiation of *the Sp7* + lineage, in which *Klf4* is a novel transcription factor.

## Introduction

The vertebrate skeleton is made of cartilage and bone, which is derived from three different embryonic lineages^1^. Cells arising in these lineages proliferate and migrate to form mesenchymal condensations^2^. Embryonic mesenchymal cells differentiate into chondrocytes in cartilage, osteoblasts, and osteoclasts in bone. Intramembranous ossification and endochondral ossification are two major methods of mesenchymal cells involved in bone formation^3,4^. In both approaches, mesenchyme-derived osteoblasts play a critical role in bone formation and, ultimately, bone remodeling, involving a complex gene regulatory network. Gene expression is orchestrated by cis-regulated elements, including enhancers and promoters, that recruit transcription factors and their cofactors^5^. Transcription factors (TFs) can bind to specific DNA motifs and lead to an on/off mode of transcriptional regulation, which eventually determines the spatial and temporal expression of genes. TFs have been reported to be key factors in stem cell pluripotency maintenance and cell fate decision^6,7^. A clear understanding of how tissue-specific TFs interact with each other and their downstream targets will provide insights into the mechanism of gene regulation in osteoblast differentiation.

Bone morphogenetic proteins (BMPs), members of the transforming growth factor beta (TGF-β) superfamily, regulate skeletal development and tissue homeostasis^8,9^ through a cascade of downstream signal transduction. As multifunctional proteins, BMPs are considered one of the key mediators in vertebrate skeletal biology. Among them, bone morphogenetic protein 2 (BMP2), a pivotal family member, has been abundantly reported as a major inducer of cartilage and bone formation^10–13^. The potent osteogenic effects of BMP2 are mediated in part by the formation of RUNX2-SMAD complexes^14,15^. In addition, BMP2 can induce SP7(Osterix) expression by *Dlx5*^16^ and induce ALPL expression in a Wnt autocrine loop^17^ in vitro. Using a conditional knockout mouse model, the function of BMP2 in different lineages has also been studied. *Sp7*-Cre mouse lines characterize early osteoblast precursors, making it easier to observe osteoblast development^18^. Previous studies using *Sp7-*Cre*; Bmp2*^fx/fx^ mice demonstrated that BMP2 can determine the specification of osteoblast cell fate^19^, long bone strength^20^, and endochondral healing^21^. It can also regulate enamel-related gene expression^22,23^ and control tooth root development^24^. These studies verified the significance of BMP2 in mineralized tissue development in vivo. However, the detailed regulatory mechanism of how BMP2 initiates its downstream transcription factors to directly determine or affect mesenchymal cell differentiation remain largely unclear.

In this study, we attempted to determine the downstream functional TFs by analyzing the enrichment of their fingerprint through changes in open chromatin regions, which are associated with cell fate decision^25–27^. Hence, we inactivated *Bmp2* in early osteoblast precursors using *Sp7*-Cre in vivo and then utilized RNA-seq and ATAC-seq to profile the target of BMP2 during osteoblast differentiation in vitro. In summary, the present study outlines a comprehensive BMP2-dependent gene regulatory network governing osteoblast differentiation of the *Sp7*+ lineage.

## Results

### Loss of *Bmp2* in *Sp7*+ lineage inhibits postnatal bone development by inhibiting osteoblastogenesis

**Fig. 1:**
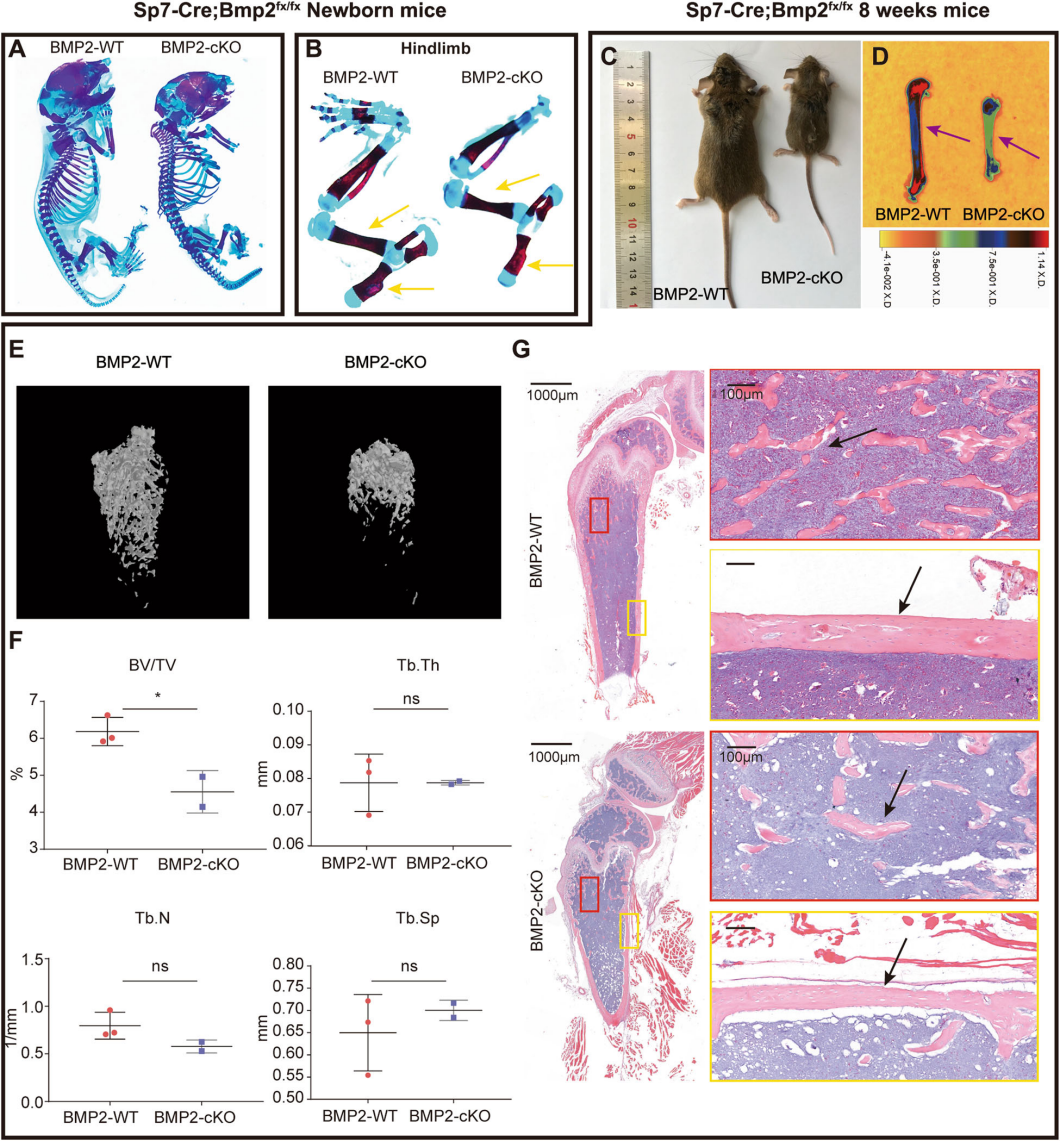
*Sp7-Cre*; *Bmp2*^fx/fx^ mice showed decreased bone mineralization. **A** Alizarin red/Alcian blue staining in newborn *Bmp2*^fx/fx^ (BMP2-WT) and *Sp7-Cre*; *Bmp2*^fx/fx^ (BMP2-cKO) mice. *n* = 3. **B** Alizarin red/Alcian blue staining of hindlimbs from newborn BMP2-WT and BMP2-cKO mice. Yellow arrowheads indicated the hindlimbs of mutant mice were thinner but normal ossified. **C** Photographic analysis of 8-week-old BMP2-WT and BMP2-cKO mice. *n* = 6. **D** Heat map of X-ray analysis of 8-week-old BMP2-WT and BMP2-cKO mice. Purple arrowheads indicated decreased ossification in the BMP2-cKO femurs. *n* = 6. **E**, **F** The micro-CT scans (**E**) and quantification (**F**) of 8-week-old BMP2-WT and BMP2-cKO mouse femurs. Quantification data indicate bone volume/tissue volume (BV/TV), trabecular number (Tb.N), trabecular thickness (Tb.Th), and trabecular separation (Tb.Sp). *n* = 3, ns = not statistically significant, *P < 0.05. **G** H&E staining of mouse femurs from 8-week-old BMP2-WT and BMP2-cKO mice. Black arrowheads indicated comparable trabecular bone number and cortical bone thickness. *n* = 3.

The *Sp7-*Cre; *Bmp2*^fx/fx^ mice (BMP2-cKO) were fertile, and newborns appeared normal. Skeletal staining at PN 0.5 suggested that there were no significant differences between the skeletons of the mutants and their wild-type siblings (Fig. 1A and S1B–E). The forelimbs (Fig. S1F) and hindlimbs (Fig. 1B) were normal in length but slightly thinner and underdeveloped.

However, as early as 2 weeks after birth, these mutant mice were smaller in size. At 8 weeks after birth, BMP2-cKO mice displayed a visibly smaller body size (Fig. 1C). The X-ray and micro-CT results showed underdeveloped long bones and decreased bone density in the mutant (Fig. 1D–F). H&E staining confirmed that the trabecular bone number and the width of cortical bone were reduced in BMP2-cKO (Fig. 1G). At around 16 weeks after birth, the BMP2-cKO mice exhibited abnormal incisors and mandibles (Fig. S1G–J). These craniofacial phenotypes were in agreement with a previous study^23^.

The detailed histological changes in BMP2-cKO mice of different ages were same like 8 weeks’ mutant mice (Fig. S2A–C). Safranin O staining showed irregularly arranged and abnormally shaped chondrocytes in the mutant (Fig. S2D).

We then asked if such defects in bone development were due to either cellular proliferation or osteoblast differentiation. In 8-week-old mice, Ki67 staining revealed reduced cellular proliferation near the growth plate and bone trabeculae. We then performed immunohistochemistry for osteogenic-related genes, SP7 and OPN, markers for preosteoblasts and differentiated osteoblasts, respectively^28^. Near the epiphysis and metaphysis of femurs, the SP7-positive and OPN-positive domains were significantly reduced in the mutant (Fig. S3A). These results indicated that *Bmp2* deficiency in the *Sp7*+ lineage resulted in a widespread reduction of cellular proliferation, which impacted osteoblast differentiation in vivo.

To confirm whether the deletion of *Bmp2* in the *Sp7*+ lineage inhibits osteoblast differentiation in vitro, we collected mBMSCs which were maintained in the osteogenic medium. Loss of *Bmp2* in BMP2-cKO cells was confirmed (Fig. S4D). In the Bmp2-WT groups, *Bmp2* was found to increase dramatically, while in the BMP2-cKO group it remained at a low level, which may be due to deletion of *Bmp2* upon osteogenic induction. We further examined the expression of osteogenesis-related genes by western blotting and qPCR. These maker genes remained at a significantly lower level in the BMP2-cKO group compared with the control (Fig. S3C–G). Alizarin red staining showed the number of calcium nodules was decreased in BMP2-cKO (Fig. S3B). Interestingly, we found that the loss of *Bmp2* also affected the ability of mBMSCs to proliferate (Fig. S4A–C) and undergo adipogenesis (Fig. S4E–G). In summary, the inactivation of *Bmp2* in the *Sp7*+ lineage impedes postnatal osteogenesis and inhibits cell proliferation, osteoblastogenesis, and adipogenesis in mBMSCs.

### Using RNA-seq and ATAC-seq reveals BMP2-denpendent gene regulatory network

**Fig. 2:**
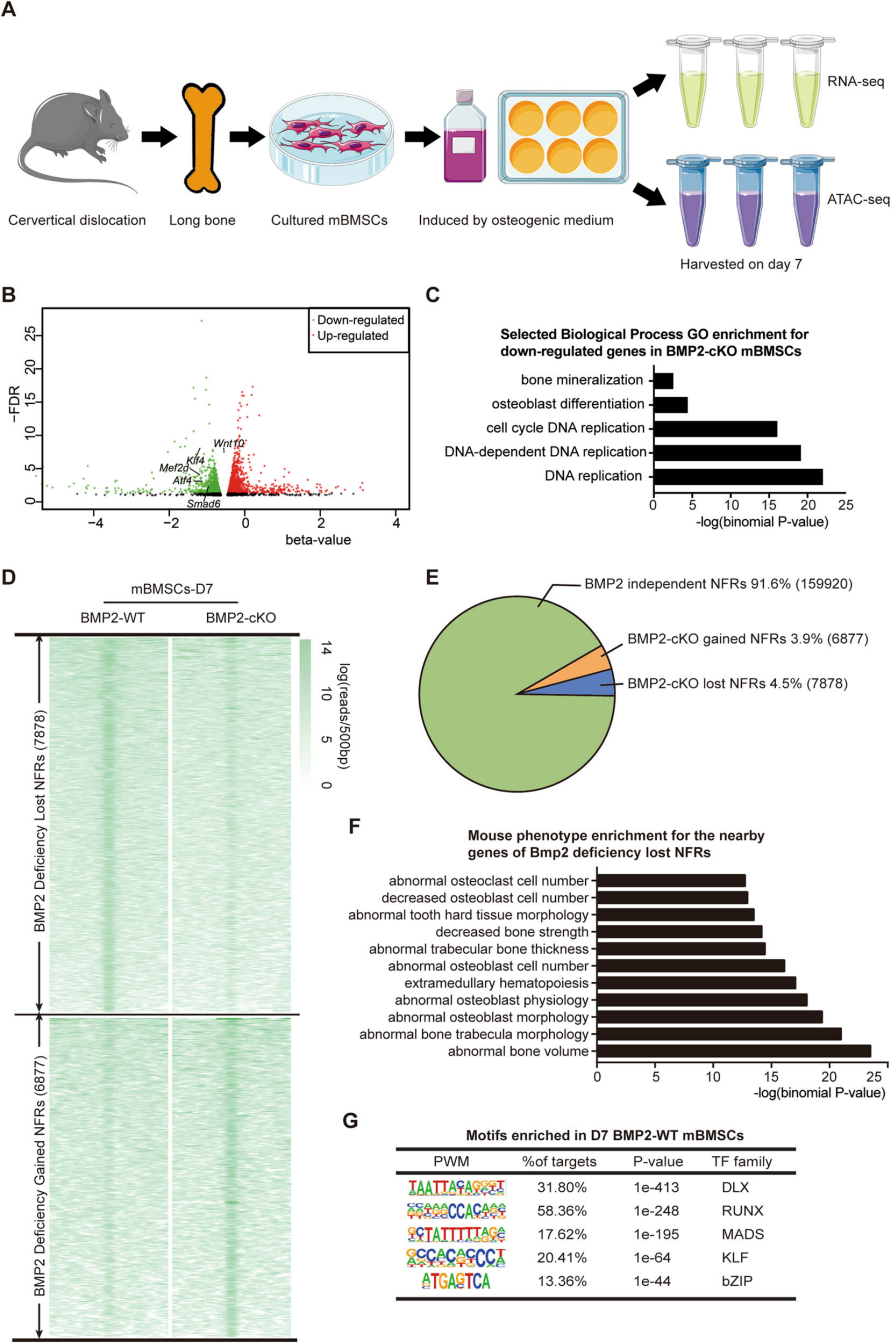
Genomewide profile of BMP2-dependent genes and open chromatin landscape in osteoblast differentiation. **A** Cell sample preparation for RNA-seq and ATAC-seq. **B** Volcano plots of RNA-seq data, showing the genes up- or downregulated in BMP2-cKO osteogenic medium induced primarily-cultured mBMSCs. Green dots indicated the downregulated genes. Red dots indicated the upregulated genes. *n* = 3. **C** GO enrichment of RNA-seq data for the downregulated genes in BMP2-cKO osteogenic medium induced primarily-cultured mBMSCs. **D** NFR summit-centred heat map of ATAC-seq signal in BMP2-WT and BMP2-cKO cells. *n* = 3. **E** Pie chart showing the distribution of all 174,675 NFRs relative to gain and loss with deficiency of *Bmp2*. **F** Mouse phenotype enrichment assay for the nearby genes of *Bmp2* deficiency lost NFRs revealed in ATAC-seq profiles. **G** Top five enriched motifs in deletion of *Bmp2* lost NFRs. PWM position weighted matrix, TF transcription factors.

To identify all potential transcription factors regulated by BMP2 in the *Sp7*+ lineage and comment on the main BMP2-related pathway(s) and transcriptional mechanism(s) during osteoblast differentiation, we utilized RNA-seq and ATAC-seq (Fig. 2A).

Based on RNA-seq results, we identified 378 genes with a significant change (*P* < 0.01 and *q* < 0.05) of expression levels due to *Bmp2* deficiency. Among them, 142 genes were significantly downregulated by *Bmp2* deficiency (*β*-value < 0), and 236 genes were upregulated. The downregulated genes were significantly enriched for mineralization-related GO terms, such as bone mineralization (GO: 0030282) and osteoblast differentiation (GO: 0001649; Fig. 2C), including *Wnt10*, *Klf4*, *Mef2d*, *Atf4*, and *Smad6* (Fig. 2B).

Next, we identified nucleosome-free regions (NFRs) from the ATAC-seq results described above, mapping them within the mouse genome. Regions of both lost- and gained- genome accessibility were observed with *Bmp2* deficiency (Fig. 2D, E). Overall, the ATAC-seq signals were enriched in putative distal regulatory regions, suggesting that abundant enhancers are involved in open chromatin accessibility (Fig. S5A, B). To identify the biological process and mouse phenotype enrichment for the nearby genes of *Bmp2* deficiency lost- and gained-NFRs, we used the GREAT algorithm. We found that *Bmp2* deficiency resulted in the loss of NFRs enriched in bone and chondrocyte development (Fig. S5C), and the relevant mice exhibited both osteoblast and osteoclast deficiencies (Fig. 2F). While *Bmp2* deficiency resulted in the gain of NFRs enriched in various biological process (Fig. S5D). In summary, by combining RNA-seq with ATAC-seq, we found that the loss of BMP2 in *Sp7*+ lineage BMSCs resulted in a decrease in the expression and accessibility of nucleosome-free regions near osteogenesis-related genes, which is responsible for the bone defects observed in BMP2-cKO.

### *Klf4* is a BMP2-dependent potential transcription factor governing osteoblast differentiation

Since nucleosome-free regions that are directly targeted by transcription factors are associated with different biological processes^29^, we investigated whether we could infer any functional TFs by analyzing the enriched motifs within the BMP2-dependent NFRs. Using HOMER, we identified the five most enriched DNA sequence motifs, corresponding to the preferred binding sites of specific transcription factors, to identify the major functional TFs of *Bmp2* in *the Sp7*+ lineage. Overall, over 80% of these cis-regulatory elements were directly targeted by these five significantly enriched motifs (Fig. 2G). In addition, we corresponded these enriched motifs with best-matched TFs according to their expression level, identified by RNA-seq. Among them, the DLX and RUNX family members ranked at the top, among which *Dlx5* and *Runx2* are critical for osteoblast differentiation^30–33^. We also observed the enrichment of the MADS and bZIP families, whose best match transcription factors, *Mef2c*^34^ and *Oasis*^35^, were expected to be meaningful TFs in osteoblast differentiation. However, BMP2-cKO gained NFRs showed different motif enrichment (Fig. S5E). Next, we used Cytoscape to build a network linking the major BMP2-WT enriched motifs and the best-matched candidate transcription factors. This network model supported the hypothesis that BMP2 regulates downstream transcription factors mainly through the Smad-dependent pathway (Fig. S6). Interestingly, we noticed that KLF motifs were highly enriched in lost NFRs. Integrated with our RNA-seq results simultaneously, we focused on a member of the KLF motif family *Klf4*, which has rarely been reported in osteoblast differentiation.

**Fig. 3:**
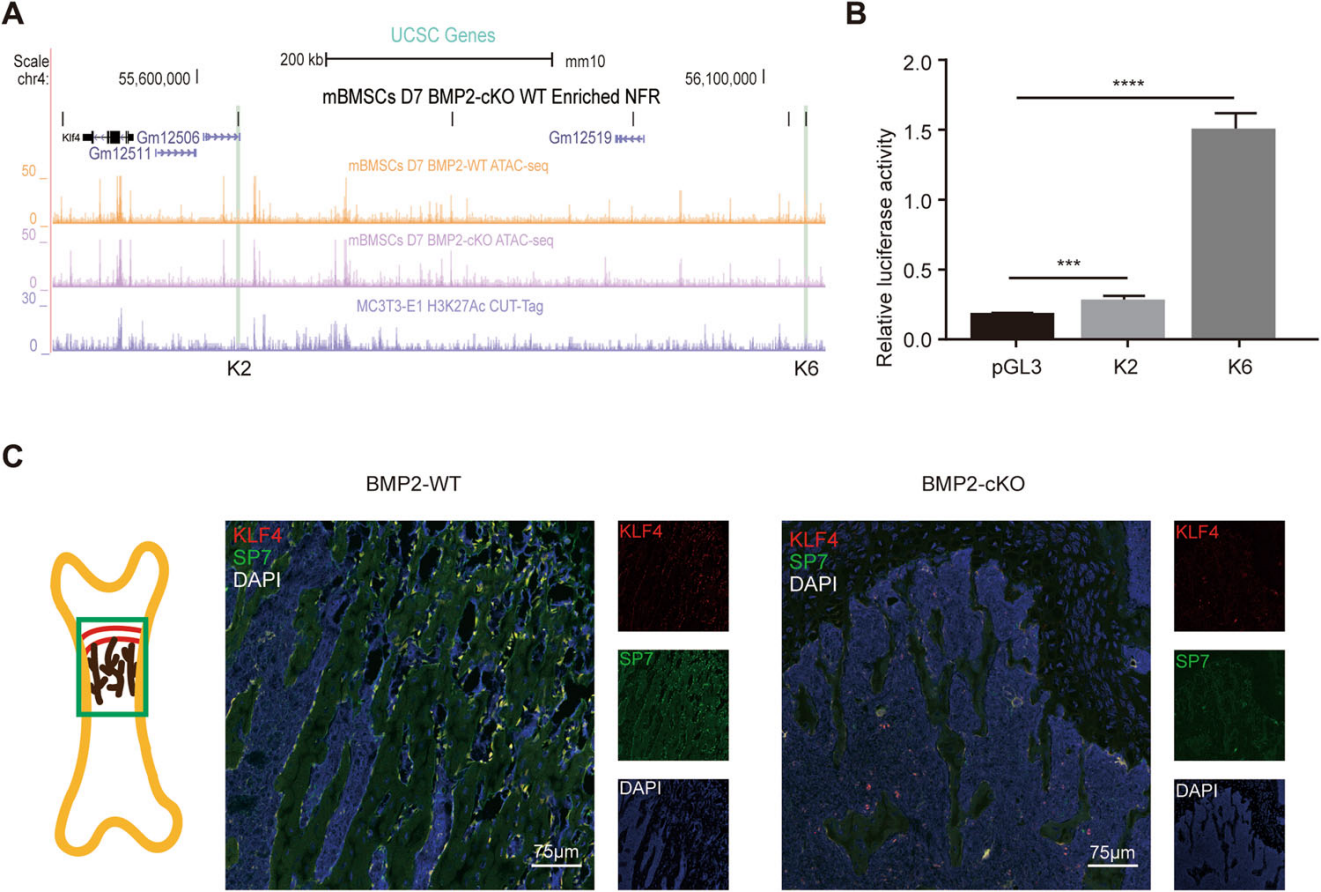
*Klf4* is a BMP2-dependent potential transcription factor governing osteoblast differentiation. **A** UCSC genome browser tracks showing representative replicate ATAC-seq and CUT&Tag at the *Klf4* locus. BMP2-dependent NFRs at Klf4 locus were named as K1 to K6. K2 and K6 are shaded in green. **B** Dual luciferase assay showing the relative enhancer activity of K2 and K6 in MC3T3-E1. *n* = 3, ***P < 0.001, ****P < 0.0001. **C** Double immunofluorescence staining of KLF4 and SP7 in 4-week-old *Bmp2*^fx/fx^ (BMP2-WT) and *Sp7-Cre*; *Bmp2*^fx/fx^ (BMP2-cKO) femurs. The image part was shown in the sketch on the left. *n* = 3.

To annotate the active enhancers/promoters among the BMP2-dependent open chromatin regions, we integrated the ATAC-seq results with the H3K27Ac CUT&Tag results from the MC3TC-E1 cell line^36^. The integration of H3K27Ac and ATAC-seq results revealed that 2338 active enhancers were BMP2-dependent in the *Sp7*+ lineage. In the “gene desert” downstream of the *Klf4* locus, we noticed several ATAC-seq peaks were “closed” in the BMP2-cKO group (Fig. 3A), indicating that BMP2 regulates *Klf4* transcription by regulating these osteoblast-associated enhancers. To evaluate whether these NFRs exert enhancer activity for *Klf4*, we chose two representative NFRs. Both were upstream of *Klf4*: K2 (mm10: chr4:55636290-55636742) and K6 (mm10: chr4:56136777-56137230). The dual luciferase activity (DLA) assay results indicated that K2 and K6 showed increased luciferase activity, with K6 showing significant fold changes (Fig. 3B).

To evaluate the change in KLF4 expression in vivo with the loss of *Bmp2*, we performed double immunofluorescence staining for KLF4 and SP7. As a result, a strong KLF4 signal was detected in the control bone trabeculae. Most of the KLF4-positive cells were SP7-positive, indicating *that* KLF4 is specifically expressed in the *Sp7*+ lineage in bone (Fig. 3C). In the BMP2-cKO mice, although the number of osteoblasts (marked by SP7) was reduced, KLF4 was barely detected in the mutant trabecular bone regions. These results suggest that *Klf4* plays a positive role as a downstream TF of BMP2 during bone development.

### Deletion of *Klf4* in *Sp7*+ lineage induces decreased bone mineralization and delayed endochondral ossification

**Fig. 4:**
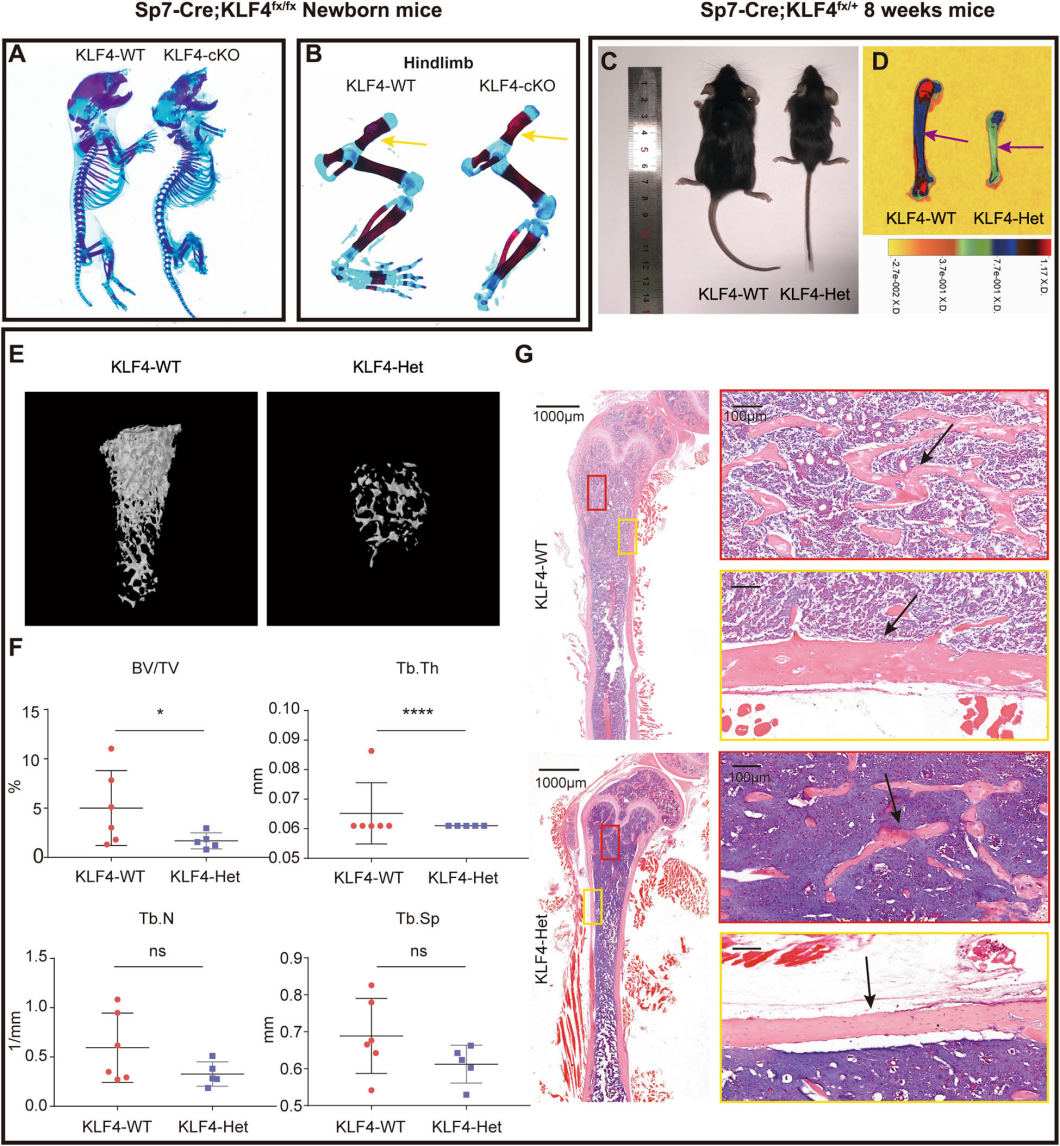
*SP7* + lineage KLF4-deficient mice suffered underdeveloped skeletons with decreased bone density. **A** Alizarin red/Alcian blue in newborn *Klf4*^fx/fx^ (KLF4-WT) and *Sp7-Cre*; *Klf4*^fx/fx^ (KLF4-cKO) mice. *n* = 3. **B** Alizarin red/Alcian blue staining of hindlimbs from newborn KLF4-WT and KLF4-cKO mice. Yellow arrowheads indicated the hindlimbs of mutant mice were shorter but normal ossified. **C** Photographic analysis of 8-week-old KLF4-WT and *Sp7-Cre*; *Klf4*^fx/+^ (KLF4-Het) mice. *n* = 6. **D** Heat map of X-ray analysis of 8-week-old KLF4-WT and KLF4-Het mice. Purple arrowheads indicated decreased ossification in the KLF4-Het mouse femurs. *n* = 6. **E**, **F** The micro-CT scans (**E**) and quantification (**F**) of 8-week-old KLF4-WT and KLF4-Het mouse femurs. Quantification data indicate bone volume/tissue volume (BV/TV), trabecular number (Tb.N), trabecular thickness (Tb.Th), and trabecular separation (Tb.Sp). *n* = 6, ns = not statistically significant, *P < 0.05, ****P < 0.0001. **G** H&E staining of mouse femurs from 8-week-old KLF4-WT and KLF4-Het mice. Black arrowheads indicated comparable trabecular bone number and cortical bone thickness. *n* = 3.

To verify our hypothesis and elucidate the role of KLF4 in vivo, we deleted *Klf4* in the *Sp7*+ lineage and found that most of the homozygotes (*Sp7-*Cre; *Klf4*^fx/fx^, KLF4-cKO) died immediately after birth. Skeletal staining at PN 0.5 confirmed decreased bone mineralization and delayed ossification in KLF4-cKO (Fig. 4A and S7B–E). The forelimbs (Fig. S7F) and hindlimbs (Fig. 4B) appeared normal in mineralization but were slightly shortened in KLF4-cKO.

Due to lethality of the homozygotes, we selected the control (*Klf4*^fx/fx^, KLF4-WT) and heterozygotes (*Sp7-*Cre; *Klf4*^fx/+^, KLF4-Het) for further study in subsequent experiments. At 8 weeks after birth, the KLF4-Het mice had smaller skeletons (Fig. 4C) and a decreased bone density (Fig. 4D–F) compared to the control littermates. H&E staining confirmed that the KLF4-Het mice also showed a reduced trabecular bone number and a thinner cortical bone (Fig. 4G). Notably, the KLF4-Het mice displayed abnormal incisors around 16 weeks after birth (Fig. S7G–J), which is similar to the craniofacial defects observed in the BMP2-cKO mice. Overall, these results suggest that KLF4 plays a role in modulating skeletal development.

At 16 weeks before birth, the KLF4-Het mice exhibited histological phenotypes similar to BMP2-cKO mice (Fig. S8A, B). At 16 weeks, the trabecular bone number, cortical bone width, and growth plate length of KLF4-Het mice showed no obvious differences compared to the KLF4*-*WT mice (Fig. S8C). Safranin O staining revealed a shortened and disorganized hypertrophic zone in the KLF4-Het mice after two weeks (Fig. S8D).

**Fig. 5:**
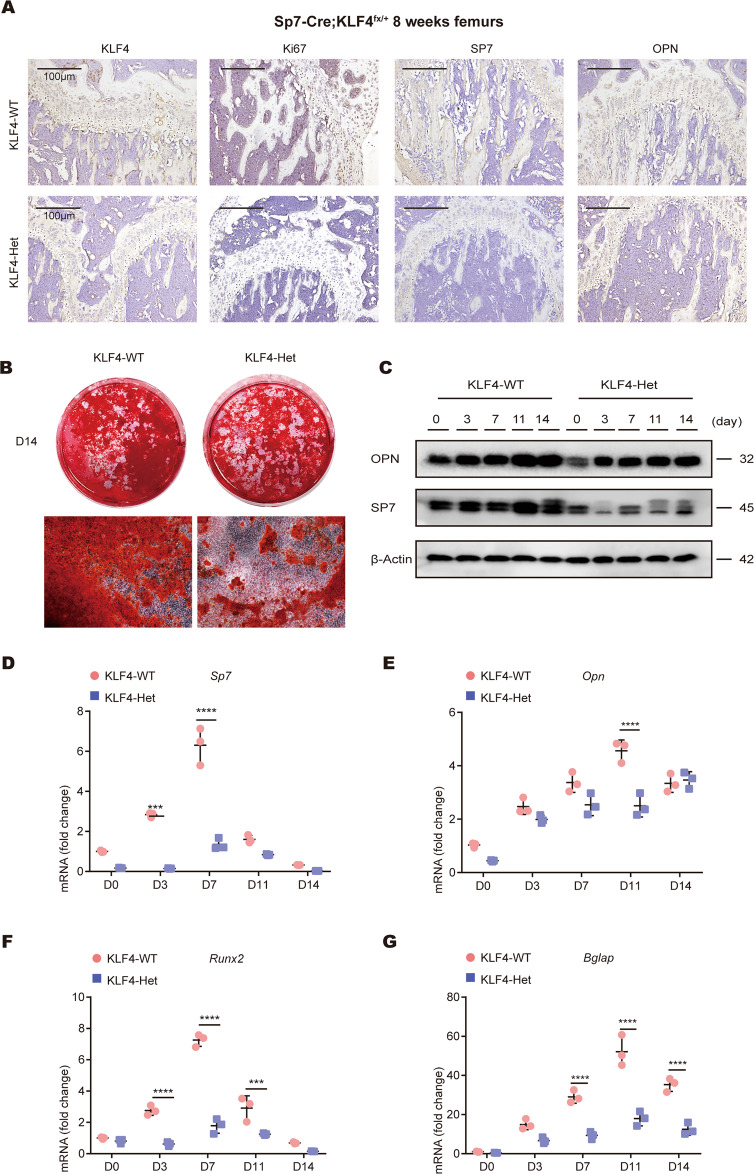
*Sp7-Cre*; *Klf4*^fx/+^ mice showed defective osteoblast formation in vivo and in vitro. **A** Immunohistochemistry (IHC) staining showed the expression of KLF4, Ki67, SP7, and OPN were decreased in 8-week-old *Sp7-Cre*; *Klf4*^fx/+^ (KLF4-Het) mouse femurs. *n* = 3. **B** mBMSCs from *Klf4*^fx/fx^ (KLF4-WT) and KLF4-Het mice were applied to Alizarin Red staining on day 14. *n* = 3. **C** Western blot analysis of SP7 and OPN protein expression levels in mBMSCs. β-Actin was used as an internal control. *n* = 3. **D**–**G** qRT-PCR analysis of *Sp7* (**D**), *Opn* (**E**), *Runx2* (**F**), and *Bglap* (**G**) mRNA expression levels in mBMSCs. *Gapdh* was used as an internal control. *n* = 3, ***P < 0.001, ****P < 0.0001.

Ki67 staining revealed that KLF4-Het mice showed reduced cell proliferation in the epiphyseal growth plate. The positive osteogenic-related markers (SP7 and OPN) domain indicated that the osteoblast number was reduced in KLF4-Het (Fig. 5A). These results demonstrate that the loss of *Klf4* in the *Sp7* + lineage impacts cellular proliferation and osteoblast differentiation in vivo. Taken together, these results indicate that KLF4 participates in osteoblast and chondrocyte maturation.

### Loss of *Klf4* in *Sp7*+ cells affects osteoblast differentiation in vitro

We then hypothesized that the deletion of *Klf4* in the *Sp7*+ lineage also inhibited osteoblast differentiation in vitro. We harvested mBMSCs and induced with osteogenic medium. *Klf4* expression in the KLF4-Het cells was confirmed (Fig. S9). During osteogenic induction, *Klf4* remained at a low level in KLF4-Het, but increased markedly in KLF4-WT. During induction, the western blotting results indicated that the SP7 and OPN protein levels were downregulated in the KLF4-Het cells (Fig. 5C). qPCR analysis revealed that the mRNA levels of *Sp7*, *Opn, Runx2*, and *Bglap* remained at a low level in the KLF4-Het cells, in agreement with the protein results (Fig. 5D–G). Alizarin red staining showed that the amount of mineralized matrix was decreased in KLF4-Het (Fig. 5B). Hence, our results indicate that the deletion of *Klf4* in *Sp7* + cells inhibits the osteoblast differentiation of mBMSCs in vitro.

### Correlating RNA-seq with ATAC-seq illustrates *Klf4* downstream gene regulatory network

**Fig. 6:**
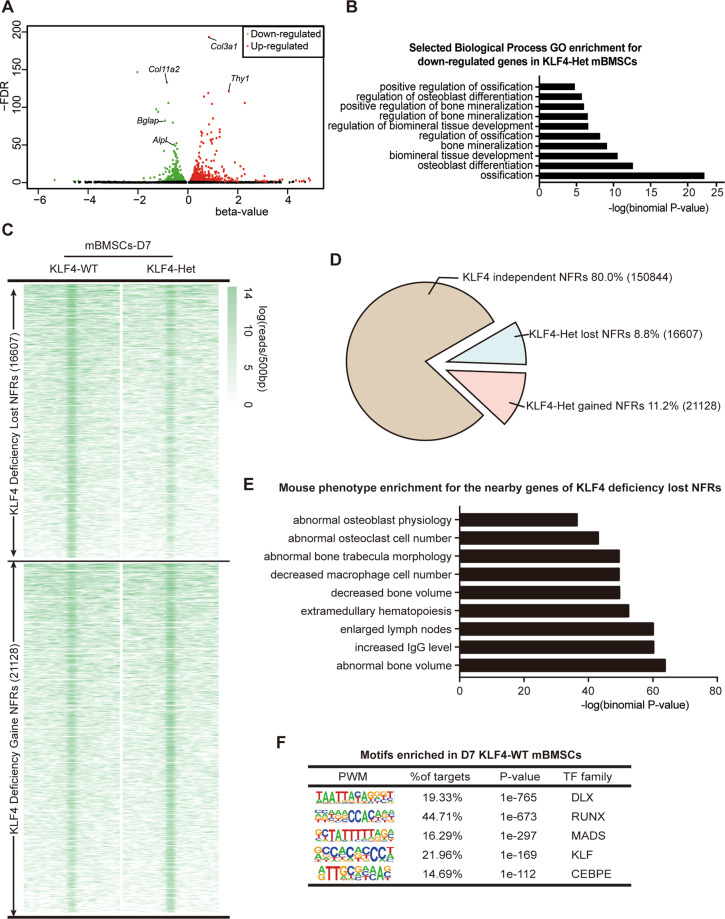
Genomewide profile of KLF4-dependent genes and open chromatin landscape in osteoblast differentiation. **A** Volcano plots of RNA-seq data, showing the genes up- or downregulated in KLF4-Het osteogenic medium induced primarily-cultured mBMSCs. Green dots indicated the downregulated genes. Red dots indicated the upregulated genes. *n* = 3. **B** GO enrichment of RNA-seq data for the downregulated genes in KLF4-Het osteogenic medium induced primarily-cultured mBMSCs revealed in RNA-seq profiles. **C** NFR summit-centred heat map of ATAC-seq signals in KLF4-WT and KLF4-Het cells. *n* = 3. **D** Pie chart showing the distribution of all 188,579 NFRs relative to gain and loss with deficiency of KLF4. **E** Mouse phenotype enrichment assay for the nearby genes of *Klf4* deficiency lost NFRs revealed in ATAC-seq profiles. **F** Top five enriched motifs in deletion of *Klf4* lost NFRs. PWM position weighted matrix, TF transcription factors.

Our KLF4-deficient transgenic mouse model supported the meaningful role of KLF4 in osteoblast differentiation. However, how KLF4 functions in osteoblast differentiation and whether it has cofactors to function as a transcriptional activator or repressor remains unknown. Using RNA-seq, we identified 1068 genes with a significant change (*P* < 0.01 and *q* < 0.05) in their expression levels due to *Klf4* deficiency. Among them, 440 genes were significantly downregulated by *Klf4* knockdown (*β*-value < 0) and 628 genes were upregulated. The representative down- or upregulated genes were annotated in our RNA-seq results (Fig. 6A). Osteogenic marker genes, such as *Alpl* and *Bglap*, were found to be significantly decreased in the mutant group. Analysis of RNA-seq results revealed that the downregulated genes were significantly enriched for mineralization-related GO terms, such as ossification (GO: 0001503) and osteoblast differentiation (GO: 0001649) (Fig. 6B).

Next, we mapped the ATAC-seq fragments within the mouse genome. Discrepancies were observed in the density of the mapped reads (Fig. 6C). We found that KLF4 was required to maintain ~8.8% (16607 NFRs) of the total NFRs in mBMSCs (Fig. 6D). On the other hand, most of *Klf4* deficiency lost- and gained- NFRs were located at the distal regions (Fig. S10A, B). We analyzed the functional annotation of the nearby genes of *Klf4* deficiency that lost NFRs and found that these NFRs were associated with metabolic-related biological processes (Fig. S10C). The corresponding mouse phenotype mainly displayed skeletal anomalies and immune response, also observed in the mice affected by *Klf4* (Fig. 6E). However, for *Klf4* deficiency, the nearby genes were enriched, including those responsible for the epithelial-to-mesenchymal transition and the negative regulation of the TGF-β signaling pathway (Fig. S10D).

We also performed motif enrichment analysis on the 16607 KLF4-dependent NFRs, and discovered five highly enriched motifs. Surprisingly, except for the CEBPE motif family, the other top four motif families were the same as those observed in BMP2-dependent NFRs (Fig. 6F). But the 21,128 gained NFRs indicated discrepant enriched motifs compared with the lost NFRs (Fig. S10E). Using Cytoscape, we built a network connecting the major KLF4-WT enriched motifs with the best-matched candidate TFs. This network model suggests that most of the downstream TFs of KLF4 were enriched in RUNX and Smad4 motifs (Fig. S11). Furthermore, we globally compared BMP2-cKO lost NFRs and KLF4-Het lost NFRs and found that 4404 NFRs overlapped between the two groups (Fig. S12A). GO enrichment assay indicated that these overlapped NFRs were enriched in skeletal system development and osteoblast differentiation (Fig. S12B). Additionally, the top four enriched TF motifs were shared among the BMP2-cKO and KLF4-Het lost NFRs groups. A different motif, ZBTB, which was previously found to regulate lymphoid development and function^37^, was also enriched for their intersected NFRs (Fig. S12C). These results indicated that KLF4 and BMP2 regulate a large set of similar NFRs through a same set of downstream TFs.

To elucidate the epistasis between BMP2 and KLF4 during osteoblast differentiation, we added BMP2 in KLF4-Het mBMSCs to determine whether BMP2 could rescue *Klf4* expression in vitro. As a result, KLF4, SP7, and OPN were found to be upregulated in KLF4-Het cells treated with BMP2 for 7 days, and KLF4 and SP7 were comparable to those in KLF4-WT (Fig. 8A). Alizarin red staining showed increased calcified matrix formation after 7 days of stimulation in KLF4-Het group with addition of BMP2 (Fig. 8B). From these results, we concluded that haploinsufficiency of KLF4 could be partially rescued by BMP2 in vitro.

The results of our integrated methods show that KLF4 regulates osteoblast differentiation with similar enriched motif as BMP2, further targeting downstream osteoblast-related genes. KLF4 was identified as a downstream member of BMP signaling.

### RUNX2 as a cofactor with KLF4 synchronously governs osteoblast differentiation

**Fig. 7:**
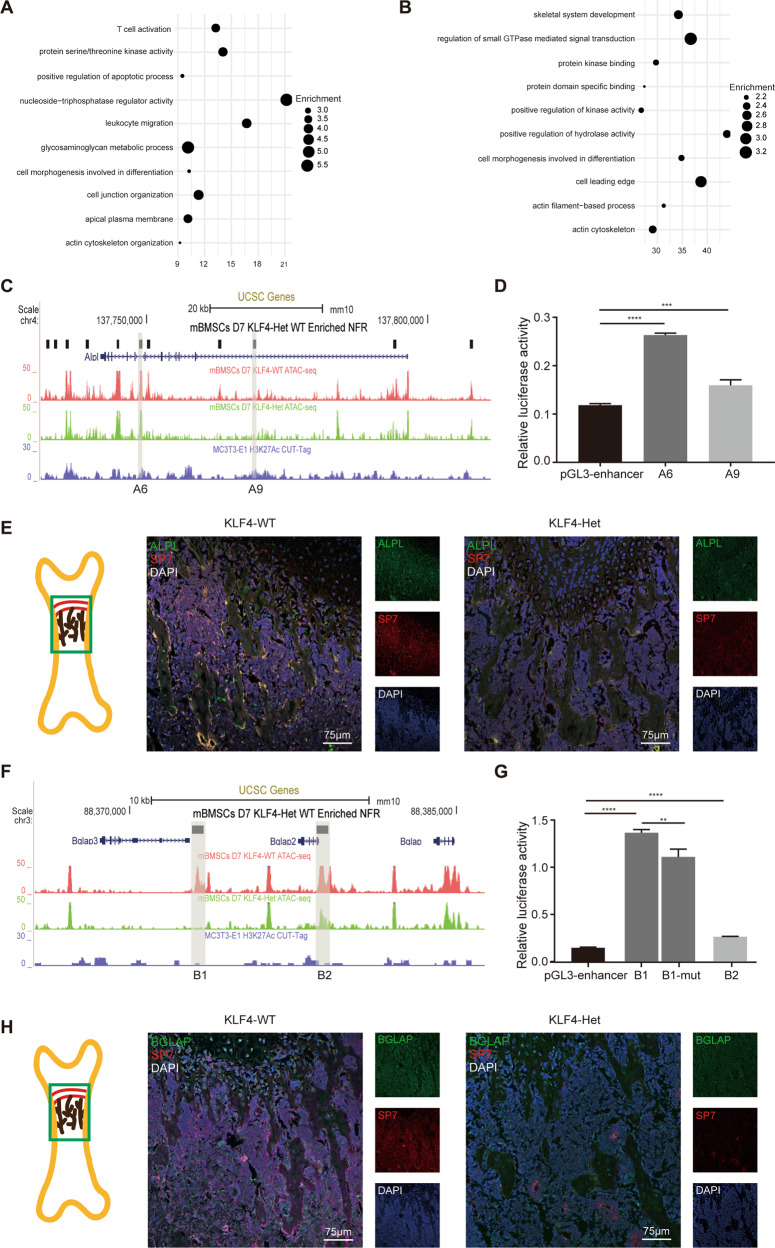
RUNX2 as a cofactor with KLF4 governing osteoblast differentiation. **A** Dot plot showing the GO enrichment assay for the nearby genes of only KLF motif occupied in KLF4 deficiency lost NFRs. **B** Dot plot showing the GO enrichment assay for the nearby genes of KLF occupied with RUNX motif in *Klf4* deficiency lost NFRs. **C** UCSC genome browser tracks showing representative replicate ATAC-seq and CUT&Tag at the *Alpl* locus. KLF4-dependent NFRs at *Alpl* locus were named as A1 to A11. A6 and A9 are shaded in gray. **D** Dual luciferase assay showing the relative enhancer activity of A6 and A9 in MC3T3-E1. n = 3, ***P < 0.001, ****P < 0.0001. **E** Double immunofluorescence staining of ALPL and SP7 in 4-week-old *Klf4*^fx/fx^ (KLF4-WT) and *Sp7-Cre*; *Klf4*^fx/+^ (KLF4-Het) femurs. The image part was shown in the sketch on the left. n = 3. **F** UCSC genome browser tracks showing representative replicate ATAC-seq and CUT&Tag at the *Bglap* locus. KLF4-dependent NFRs at *Bglap* locus were named as B1 to B2. B1 and B2 are shaded in gray. **G** Dual luciferase assay showing the relative enhancer activity of B1, B2 and B1 without KLF motif in MC3T3-E1. n = 3, **P < 0.01, ****P < 0.0001. **H** Double immunofluorescence staining of BGLAP and SP7 in 4-week-old KLF4-WT and KLF4-Het femurs. The image part was shown in the sketch on the left. n = 3.

Based on motif enrichment analysis, the *Klf4* deficiency lost NFR-enriched motifs was critical for osteoblast differentiation. Next, we investigated whether these members could regulate different or the same subset of osteogenesis genes dependent on KLF4. Hence, we examined the enrichment of the genes near the NFRs occupied with KLF motif only or a combination of KLF and other motifs. When only the KLF motif was occupied, biological processes were not associated with skeletal system development (Fig. 7A). However, when KLF was “cobound” with RUNX, DLX, MADS, or CEBPE motifs, most of the nearby genes of the related NFRs manifested significant enrichment in skeletal system development (Figs. 7B and S13A–C). These results suggest that *Klf4* itself does not directly regulate skeletal development and requires other osteogenesis-related transcription factors as cofactors to drive skeletal development.

Because the RUNX motifs accounted for 44.17% of the motifs directly affected by KLF4 in mBMSCs, and previous studies have found that KLF4 is physically associated with RUNX2^38,39^, we evaluated their physical binding results (Fig. S13D). Therefore, we focused on the NFRs with both KLF- and RUNX-motif-relevant genes. Considering *the* KLF4-deficient RNA-seq results, *Alpl* and *Bglap* were selected as downstream targets. And we chose two emblematic NFRs from the two loci separately to verify their transcription activity (Fig. 7C, F). For the *Alpl* locus, both representative NFRs were found on the *Alpl* gene sequence A6 (mm10: chr4:137748653-137749194) and A9 (mm10: chr4:137768825-137769369). The DLA assay results indicated that A6 and A9 exhibited increased luciferase activity with no obvious fold changes (Fig. 7D). Double immunofluorescence staining showed a strong ALPL and SP7 signal in the KLF4-WT bone trabeculae, but a weak ALPL and SP7 signal in the KLF4-Het group (Fig. 7E). For the *Bglap* locus, we chose B1 (mm10: chr3: 88372858-88373399) and B2 (mm10: chr3:88378577-88379119). We found that both B1 and B2 exhibited increased luciferase activity, and B1 exhibited at least a 10-fold change. We evaluated B1 as a functional enhancer in the presence of KLF motifs and found that the deletion of KLF motifs in B1 reduced enhancer activity (Fig. 7G). Collectively, these results indicate that KLF motifs are essential for *Bglap* enhancer activity. Double immunofluorescence staining showed a strong BGLAP signal in the KLF4-WT bone trabeculae, and the BGLAP-positive region was larger than SP7-positive. However, both the BGLAP- and SP7-positive regions were reduced in KLF4-Het bone (Fig. 7H). Taken together, our results suggested that KLF4 could “cobond” with Runx2 to further regulate downstream genes, including *Alpl* and *Bglap*.

## Discussion

To illustrate the BMP2-dependent gene regulatory network in osteoblast differentiation, we selectively ablated *Bmp2* in *Sp7*+ lineage in vivo and then analyzed its function in vitro. We found *Bmp2* in *Sp7*+ cells was dispensable for embryonic skeletal development but essential for postnatal endochondral ossification. Our findings are consistent with those of previous studies^12,40,41^. Due to the inhibition of postnatal bone development via changes to osteoblastogenesis, we analyzed the critical downstream TFs and focused on the function of *Klf4* in osteoblast differentiation, and our findings highlighted *Klf4* as a novel transcription factor in bone development.

KLF4 is known for its role in the maintenance of stemness in pluripotent stem cells^42^. KLF4 also functions in various cell differentiation processes, such as epithelial differentiation^43^ and adipogenesis^44^. Interestingly, our group has spent many years studying the role of KLF4 in odontoblast differentiation. Recently, we deleted Klf4 in odontoblast cells with *Wnt1*-Cre and found that *Klf4* deficiency affected dentin deposition and formation^45^. In the present study, we specifically deleted *Klf4* in the *Sp7*+ lineage to study the in vivo role of KLF4. Unexpectedly, the deletion of *Klf4* in the *Sp7*+ lineage resulted in postnatal death. As such, we selected heterozygotes as our main targets. KLF4-Het mice exhibited osteogenesis defects analogous to those observed in BMP2-cKO mice, indicating that KLF4 are necessary in osteoblasts. Our RNA-seq and ATAC-seq results also elucidated the functions of KLF4 in bone formation. We reported KLF4 itself was not sufficient to prime osteoblast differentiation, but rather acted with RUNX2 as a cofactor to activate potential enhancers and regulate downstream genes, to determine osteoblast differentiation and osteogenesis. Our results, therefore, markedly supported the multidisciplinary perspective of BMP signaling and established unique insights into the role of KLF4 during bone development.

The current study presents some controversial results compared to similar previous studies. A group culturing mBMSCs from BMP2-WT and BMP2-cKO mice in osteogenic medium suggested that *Bmp2* is dispensable for osteoblast differentiation in the *Sp7*+ lineage^19^. However, our results indicate opposite conclusion. This distinction may be due to differences in the composition of the osteogenic medium. In the present study, 10 nmol/L of dexamethasone (Dex) was added to the osteogenic medium, while other studies did not. Dexamethasone promoted the expression of osteogenic markers, such as RUNX2, SP7, and OPN in vitro^46–49^. The deletion of *Bmp2* in the *Sp7*+ lineage may block the activation of Dex-induced osteo-related signaling pathways. In addition, using *Col1α1-*Cre*; Bmp2*^fx/fx^ mice, other researchers demonstrated that the *Bmp2* in osteoblasts is required for terminal differentiation^50^. It is worth noting that *Klf4* is a controversial transcription factor. A previous study reported that KLF4 is a negative regulator of osteoblast differentiation in vitro and in vivo^39^, using *Col1α-*Cre; *Klf4*^fx/fx^ mice. Using different Cre mouse lines, we obtained different conclusions about KLF4. *Col1α1-*Cre mainly targets mature osteoblasts, while *Sp7-*Cre mainly targets immature osteoblasts^51^. These results suggest that, during embryogenesis and postnatal bone development, KLF4 plays temporal- and spatial-specific roles in the process of bone development. However, we were unable to elucidate the precise function of KLF4 during the whole cell differentiation process. Based on varied conditions in vivo, it may activate tissue-specific enhancers and perform a spatial-temporal function.

Members of the BMP superfamily affect almost all aspects of bone, cartilage, and joint biology. Defective BMP superfamily molecules are the underlying cause of human skeletal pathologies. Thus, modulating BMP superfamily signaling is a potential method for inducing stem cells for tissue repair. KLF4-Het mice exhibited the consistency of the long bone phenotype like BMP2-cKO mice, but chondrocyte defects were milder. In addition, KLF4-cKO mice exhibited more significant cranial dysplasia. Although our results showed that *Klf4* was controlled by BMP signaling, other key signaling pathways must participate to direct the regulation of *Klf4* in skeletal development. Further study is necessary to determine the branched regulatory network in the process of endochondral ossification and intramembranous ossification, which may provide new therapeutic approaches for skeletal system disease.

**Fig. 8:**
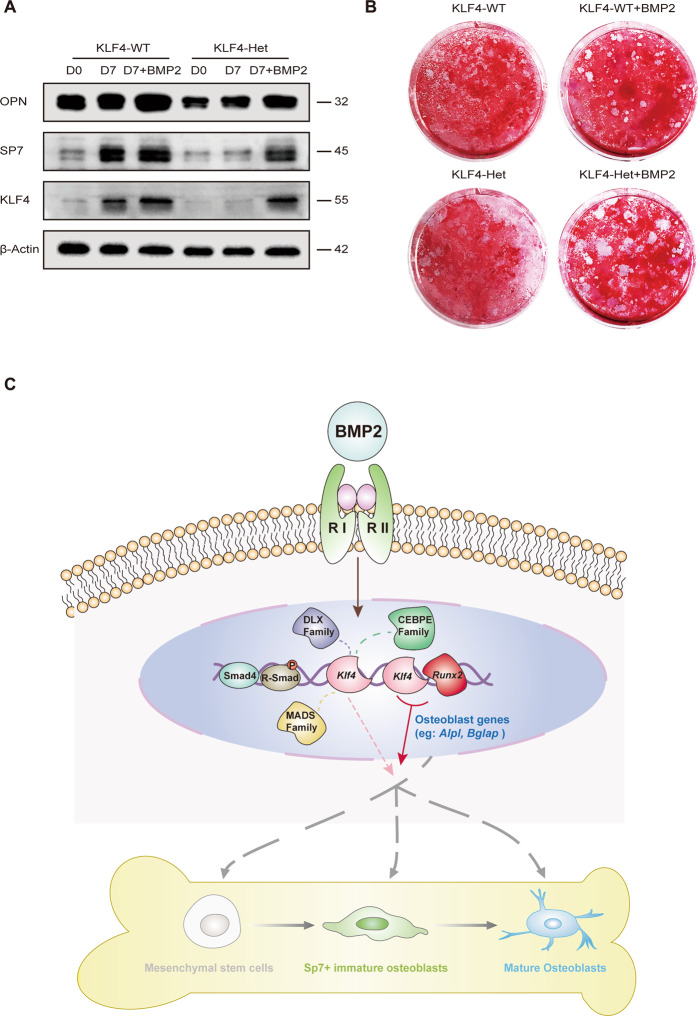
*Klf4* is a BMP2-dependent transcription factor governing osteoblast differentiation. **A** Western blot analysis of KLF4, SP7, and OPN protein expression levels in osteogenic medium induced primarily-cultured mBMSCs with or without BMP2 treatment. β-Actin was used as an internal control. *n* = 3. **B** BMSCs from *Klf4*^fx/fx^ (KLF4-WT) and *Sp7-Cre*; *Klf4*^fx/+^ (KLF4-Het) mice with or without BMP2 treatment were applied to Alizarin Red staining on day 7. *n* = 3. **C** During the osteoblast differentiation, BMP superfamily signal molecules act locally on target cells in which targeted transcription factors switch is turned on*. Klf4* as a downstream transcription factor involved during mesenchymal stem cells to mature osteoblasts differentiation. *Klf4* itself could not prime whole differentiation process, but it could interact with coactivators such as RUNX2 (or DLX, MADS, CEBPE family TFs) to initiate the expression of osteogenic marker genes (eg: *Alpl* and *Bglap*) further governing osteogenesis and osteoblast maturation.

To summarize, we propose *Klf4* as a BMP2-dependent novel transcription factor in osteoblast differentiation (Fig. 8C). When the extracellular signal molecule BMP2 binds to the transmembrane type I and type II receptors, the BMP signaling pathway is activated. BMP signaling synergizes osteogenic transcription factors, such as *Runx2* and *Klf4*. KLF4 itself was not able to prime osteoblast differentiation but could “cobound” with osteogenesis-enriched TFs (RUNX, DLX, MADS, and CEBPE) to regulate osteoblast differentiation. Among them, RUNX2, a coactivator with KLF4, regulates the expression of *Alpl* and *Bglap*. Collectively, our findings indicate the existence of a genome-wide BMP2-dependent gene regulatory network and provide novel insights into the role of KLF4 in osteoblast differentiation.

## Materials and methods

### Transgenic mice generation and maintenance

All mouse-related experimental protocols were approved by the Institutional Animal Care and Use Committees at the School and Hospital of Stomatology of Wuhan University (protocol no. S07917110B). B6;129S4-Bmp2^tm1Jfm^/J (hereafter *Bmp2*^fx/fx^) mice^23,52^ and B6.Cg-Tg(Sp7-tTA,tetO-EGFP/cre)1Amc/J (hereafter *Sp7-*Cre) mice^18^ were purchased from Jackson Laboratory, B6.129S6-Klf4^tm1Khk^/Mmmh (*Klf4*^fx/+^) mice^53^ were purchased from the Mutant Mouse Regional Resource Centers (MMRRC). To rule out potential defects in *Sp7*-Cre mice as previously reported^54^, we also examined every *Sp7*-Cre littermate. All mice were maintained with good ventilation and kept on a standard diet and a 12-h day/night rhythm.

### Immunohistochemistry

The 6-μm paraffin sections were deparaffinized, rehydrated, and antigen-retrieved with gastric enzyme. The sections were incubated with anti-BMP2 (ab6285; Abcam, Cambridge, MA, USA), anti-SP7 (sc-393325; Santa Cruz Biotechnology, Santa Cruz, CA, USA), anti-OPN (0806-6; HuaAn Biotech, Hangzhou, Zhejiang, China), anti-KLF4 (11880-1-AP; Proteintech, Wuhan, Hubei, China), and anti-Ki67 (ET1609-34; HuaAn Biotech) overnight at 4 °C. After incubation with horseradish peroxidase-conjugated secondary antibody, the sections were visualized using a diaminobenzidine substrate kit (DAKO, Carpinteria, CA, USA). The immunostained sections were counterstained with hematoxylin.

### Double immunofluorescence staining

The 6-μm paraffin sections were deparaffinized, rehydrated, antigen-retrieved, blocked, and then incubated with primary antibodies overnight at 4 °C. The antibodies included anti-KLF4, anti-SP7, anti-BGLAP (ab93876; Abcam), and anti-ALPL (AF2910; R&D Systems, Minneapolis, MN, USA). The sections were then incubated with Alexa Fluor 488- or 594-conjugated secondary antibodies (Jackson Immuno Research, West Grove, PA, USA). Finally, the stained sections were mounted with DAPI.

### Quantitative real-time PCR assay

Total RNA from cultured cells was isolated using HP Total RNA kit (Omega Bio-Tech, Norcross, Georgia, USA). Reverse transcription was performed using RevertAid RT Reverse Transcription Kits (Thermo Fisher Scientific, Waltham, MA, USA). Quantitative real-time PCR (qRT-PCR) was performed using the CFX96 Touch Real-Time PCR Detection PCR System (BIO-RAD, Berkeley, CA, USA) with SYBR Premix Ex Taq (Takara, Kusatsu, Gunma, Japan).

### Western blot analysis

Cells were lysed in a buffer containing protease inhibitors. Total proteins were harvested and equal amounts of protein were resolved by 12% SDS/PAGE and then transferred onto a PVDF membrane. The membranes were incubated with the primary antibody for SP7, OPN, KLF4 (ab106629; Abcam), or β-Actin (PM053; MBL, Nagoya, Aichi-ken, Japan) at 4°C overnight. After incubation with horseradish peroxidase-labeled IgG. ECL solution (Pierce Biotechnology, Rockford, IL, USA) was used for signal visualization.

### RNA-seq library generation

Cultured mBMSCs were induced with osteogenic medium for 7 days, and 3 µg of total RNA was isolated using an RNeasy mini kit (Qiagen, Valencia, CA, USA). Genomic DNA was digested using Turbo-DNaseI using DNasel (Promega, San Luis Obispo, CA, USA). The library quality was assessed using a DNA 1000 kit (5067–1504; Agilent) on a Bioanalyzer 2100 (Agilent, Santa Clara, CA, USA). NEBNext Ultra RNA Library Prep Kit (New England Biolabs, Ipswich, MA, USA) was used to generate and index RNA-seq libraries. 150-bp-paired-end sequencing was performed using a Hiseq X Ten sequencer (Illumina, San Diego, CA, USA, provided by Annoroad Genomics Company (China)). Then, sequencing reads were pseudo-aligned to the mm10 mouse genome.

### ATAC-Seq library preparation

Library preparation was performed using our previously reported protocol^45^. Briefly, mBMSCs were cultured in osteogenic medium for 7 days, and ~50,000 cells were used for the library. Cells were washed in cold PBS, resuspended in cold lysis buffer (10 mM Tris-HCl at pH 7.4, 10 mM NaCl, 3 mM MgCl_2_, 0.1% NP-40). The tagmentation reaction was performed using Tn5 transposase (TD501; Vazyme, Nanjing, Jiangsu, China). The DNA library was eluted using a purification kit (Mini Elute kit; Qiagen) to obtain purified DNA, which was amplified and barcoded with the NEBNext High-Fidelity 2× PCR Master Mix (New England Biolabs). DNA fragments were purified using AMPure beads (Beckman Coulter, Brea, CA, USA), and the library quality was analyzed using BioAnalyzer 2100. Sequencing was performed using a HiSeq X Ten sequencer (Illumina, provided by Annoroad Genomics Company (China)).

### Plasmid constructs and dual luciferase report assay

All candidate enhancer elements described were cloned using mouse genomic DNA. Wild-type or mutant DNA fragments were cloned into the pGL3-basic plasmids and Sangon sequencing was performed to validate the sequence. MC3T3-E1 cells were seeded in 24-well plates and transfected with 1 μg of reporter luciferase vector and 200 ng of pRL-TK using Lipofectamine 2000 reagent (Invitrogen). After 48 h of transfection, the cells were harvested and the luciferase activity was measured using the Dual-Luciferase Reporter Assay System (Promega), according to the manufacturer’s instructions. GloMax 20/20 Luminometer (Promega) was used to evaluate the ratio between firefly and Renilla activity.

### Sample processing

Each experiment was performed at least three independent replicates. All data represented biological replicates. Sample size of animals was at least three based on preliminary tests. Wild-type samples were collected randomly and conditional knockout samples were collected based on obvious phenotype deficiency. No samples were excluded from the analyzation. Data was analyzed blind.

### Statistical analysis

Except for the high-throughput sequencing results, data are presented as the mean ± standard deviation (SD; *n* > 3). One-way analysis of variance (ANOVA) was used to compare multiple group comparisons, and Student’s t test was applied to compare two groups. *P* < 0.05 was considered statistically significant.

## Supplementary information

Supplementary File

Suppl figure 1

Suppl figure 2

Suppl figure 3

Suppl figure 4

Suppl figure 5

Suppl figure 6

Suppl figure 7

Suppl figure 8

Suppl figure 9

Suppl figure 10

Suppl figure 11

Suppl figure 12

Suppl figure 13

## Data Availability

All sequencing profiles, including RNA-seq and ATAC-seq, were uploaded to the China National Center for Bioinformation (CNCB). The datasets are available in the GSA (accession NO. CRA003341).
