# Supplementary material for: BMP2-dependent gene regulatory network analysis reveals Klf4 as a novel transcription factor of osteoblast differentiation
Source: Cell Death Dis. 2021 Feb 19;12(2):197. doi: 10.1038/s41419-021-03480-7 (PMC7895980; doi:10.1038/s41419-021-03480-7)
Supplement: Supplementary file 1 — Supplementary File [file 41419_2021_3480_MOESM1_ESM.docx]

**1. Materials and Methods**

**1.1 Whole mount Alizarin red and Alcian blue staining**

The genotypes of transgenic mice were subjected to polymerase chain reaction (PCR) using tail DNA **(Fig. S1A and Fig. S7A)**. Both male and female mice were used without distinction in all analyses. For skeletal preparation, mice were first skinned in hot tap water for 20–30 s at 65°C. The eyes, all skin, internal organs, adipose tissue, and bubbles from the body cavity were removed. Next, the embryos were fixed in 95% ethanol overnight at room temperature, and then in acetone for 24 h. The mice were stained with Alizarin red (Sigma-Aldrich, St Louis, MO, USA) and/or Alcian blue solutions (Sigma-Aldrich) with 10% glacial acetic acid and 70% ethanol for 3 days at 37°C. Finally, the mice were washed in 1% KOH for 2 to 4 days. After staining, the cartilages were characterized by blue staining and mineralized bone was characterized by red staining.

**1.2 Radiographic procedures**

For X-ray analysis, radiography was performed using a radio system (In-Vivo FX PRO; Bruker, Billerica, MA, USA) at 26 kV. For micro-CT analysis, the morphology of the bone, the trabecular bone volume/tissue volume ratio (BV/TV: %), trabecular thickness (Tb.Th: m), trabecular number (Tb.N: mm^-1^), and trabecular bone separation (Tb.Sp: m) were recorded using a micro-CT machine (70 kV, 114 µA, 8 w, 200 ms, step=10.5 µm) (SkyScan1176; Bruker) and analyzed by CTAn (version 1.18.8.01) (Bruker).

**1.3 Histology and tissue preparation**

All mouse femurs from control and mutant mice were harvested, skinned, and eviscerated before fixing in 4% PFA at 4°C overnight. Samples were then decalcified in 10% EDTA for 2-4 weeks, followed by dehydration using a graded ethanol series and paraffin embedding. For paraffin sections, samples were dehydrated in graded ethanol series, cleared in xylene, embedded in paraffin, sectioned at 6-μm thickness with a Leica microtome (Wetzlar, Hesse-Darmstadt, Germany), and mounted on SuperFrost™ Plus slides (Thermo Fisher Scientific, Waltham, MA, USA). The prepared sections were analyzed for H&E staining, safranin O staining, and immunostaining.

**1.4 Cell culture**

Primary mouse bone marrow stromal cells (mBMSCs) were isolated from the bone marrow cells of the femora of 4-8-week-old mice (male or female) and maintained in α-MEM (Hyclone, Logan, UT, USA) containing 10% fetal bovine serum (FBS) (Gibco, Waltham, MA, USA), penicillin (100 U/mL), and streptomycin (100 μg/mL) in a 37°C incubator containing 5% CO^2^. When cells reached 80-85% confluence, the mBMSCs were subjected to induction by osteogenic medium and treated with 100 ng/ml BMP2 (355-BM-010; R&D Systems) at the indicated time. Primary mouse BMSCs were harvested for the extraction of RNA and protein. Osteogenic medium was comprised of 50 µg/mL of ascorbic acid (Sigma-Aldrich), 10 mmol/L of sodium β-glycerophosphate (Sigma-Aldrich), and 10 nmol/L of dexamethasone (Sigma-Aldrich). Fixed cells were stained with 2% Alizarin Red S (Sigma-Aldrich) at pH 4.2 to evaluate the cell matrix mineralization.

**1.5 CUT&Tag library preparation**

To generate the CUT&Tag library, Hyperactive^TM^ In-Situ ChIP Library Prep Kit for Illumina (TD902; Vazyme) was used according to the manufacturer’s protocol. Briefly, approximately 10,000 MC3T3-E1 cells in each biological replicate were harvested and centrifuged at 600 × *g* at room temperature for 3 min, and then washed with 1 mL of wash buffer (TD902; Vazyme) supplemented with protease inhibitors (Roche Complete Protease Inhibitor EDTA-Free Tablet; Sigma-Aldrich). The supernatant was removed, and cells were resuspended in 100 µl of wash buffer per sample. Then, 10 µl of Concanavalin A-coated magnetic beads (TD902; Vazyme) per sample were washed twice in 100 µl of binding buffer (TD902; Vazyme) using a magnet stand and resuspended in 10 µl of binding buffer. Then, the beads were added to the cells with gentle vortexing and incubated with rotation for 10 min at room temperature. The unbound supernatant was removed, and bead-bound cells were resuspended in 50 µL of pre-cooled antibody buffer per tube (2 mM EDTA, 0.1% BSA in Dig-wash Buffer (0.05% Digitonin in Wash Buffer)) before incubating at a dilution of 1:50 in primary antibody against H3K27ac (ab4729; Abcam) with rotation overnight at 4°C. Rabbit IgG (AC005; Abclonal, Wuhan, Hubei, China) was used as the negative control. After removing the primary antibody on the magnet stand, a secondary antibody (Guinea Pig anti-Rabbit IgG antibody) (611-201-122; Rockland Immunochemicals, Philadelphia, PA, USA) was diluted 1:100 in 50 µl of Dig-Wash buffer and incubated with the cells at room temperature for 1 h. The cells were washed three times in 0.8 ml Dig-Wash buffer using a magnet stand to remove unbound antibodies. A dilution of Hyperactive pA-Tn5 Transposon complex (0.04 µM) was prepared in Dig-300 Buffer (TD902; Vazyme) supplemented with 0.01% digitonin and protease inhibitors (Roche Complete Protease Inhibitor EDTA-Free Tablet; Sigma-Aldrich). The cells were then incubated with 100 µl of pA-Tn5 Transposon complex with rotation at room temperature for 1 h. The cells were washed three times in 0.8 ml of Dig-300 buffer using a magnet stand to remove unbound pA-Tn5 transposon. Next, the cells were resuspended in 300 µL of tagmentation buffer (10 mM MgCl_2_ in Dig-300 Buffer) and incubated at 37°C for 1 h. To stop the tagmentation reaction, 10 µL of 0.5 M EDTA, 3 µl of 10% SDS, and 2.5 µL of 20 mg/mL proteinase K were added to each sample and incubated overnight at 37°C. The DNA was purified, amplified, and indexed using the NEBNext High-Fidelity 2× PCR Master Mix (New England Biolabs). The VAHTS DNA clean beads (N411; Vazyme) were used to purify the library. Sequencing was performed using a HiSeq X Ten sequencer (Illumina, provided by Annoroad Genomics Company (China)) with a sequencing depth of 6G bases for each sample.

**1.6 Cell proliferation assay**

Cell proliferation was examined using a 5-ethynyl-2-deoxyuridine (EdU, RiboBio, Guangzhou, Guangdong, China) incorporation assay and cell counting kit-8 (CCK-8; Dojindo, Kumamoto, Kyushu, Japan) assay. For the EdU assay, the control and mutant cells were incubated in 50 µM EdU agent for 3 h at 37°C, according to the manufacturer’s protocol. The observation and calculation of EdU-positive cells were performed using fluorescence microscopy. For the CCK-8 assay, mBMSCs were seeded into 96-well plates and maintained in normal culture medium for different times (1, 3, 5, 7, 9, and 11 days). After different days of culture (day 1, 3, 5, 7, 9, and 11), 10 µL of CCK-8 per 100 µL of culture medium was added to each well. After incubation at 37°C for 1 h, the optical density (OD) of each well was read at a wave-length of 450 nm, according to the manufacturer’s instructions.

**1.7** **Immunoprecipitation assay**

MC3T3-E1 cells were harvested and lysed. Immunoprecipitation was performed by incubating the precleared lysates with Anti-RUNX2 (12556S; CST, Boston, MA, USA) and rabbit control IgG (AC005; Abcam) at 4°C overnight. Protein A/G magnetic beads (Bimake, Houston, Texas, USA) were added and rotated at 37°C for 1 h. The immunocomplexes were harvested using DynaMag-Spin magnets (Thermo Fisher Scientific). The pellets were washed three times with binding buffer at 4°C for 5 min. Proteins were denatured by SDS-PAGE and examined by western blot analysis with the anti-KLF4 (11880-1-AP; Proteintech) and anti-RUNX2 antibodies.

**2. Supplementary Figure Legends**

**Supplementary Fig. 1.** *Sp7-Cre*; *Bmp2*^fx/fx^ mice exhibited defective calvaria and mandible phenotype.

(A) All mice were genotyped by PCR from tail snip DNA. (B, C) The skulls (B) and ribs (C) displayed normal size and ossification in the *Sp7-Cre*; *Bmp2*^fx/fx^ (BMP2-cKO) mice. n=3. (D, E) The clavicles (D) and mandibles (E) were smaller and undercalcified in the BMP2-cKO mice. n=3. (F) The forelimbs were thinner but normal ossified in the BMP2-cKO mice. n=3. (G) Tooth photographic analysis of 16-week-old *Bmp2*^fx/fx^ (BMP2-WT) and BMP2-cKO mice. Black arrowheads indicated incisors were asymmetric with open forked in BMP2-cKO mice compared with BMP2-WT. n=6. (H-I) Stereo Microscope analysis (H) and high magnification (I, J) of mandibles from 16-week-old BMP2-WT and BMP2-cKO mice. Stereo Microscope analysis indicated defective mandibles and caries in the BMP2-cKO mice. n=6.

**Supplementary Fig. 2.** Histology analysis of *Bmp2*^fx/fx^ and *Sp7-Cre*; *Bmp2*^fx/fx^ mice at different ages.

(A-C) H&E staining of mouse femurs from 2-week-old (A), 4-week-old (B) and 16-week-old (C) *Bmp2*^fx/fx^ (BMP2-WT) and *Sp7-Cre*; *Bmp2*^fx/fx^ (BMP2-cKO) mice. n=3. (D) Safranin O staining of mouse femurs from 2-week-old, 4-week-old, 8-week-old and 16-week-old BMP2-WT and BMP2-cKO mice. n=3.

**Supplementary Fig. 3.** *Sp7-Cre*; *Bmp2*^fx/fx^ mice showed defective osteoblast formation *in* *vivo* and *in vitro*.

(A) Immunohistochemistry (IHC) staining showed the expression of BMP2, Ki67, SP7 and OPN were decreased in 8-week-old *Sp7-Cre*; *Bmp2*^fx/fx^ (BMP2-cKO) mouse femurs. n=3. (B) mBMSCs from *Bmp2*^fx/fx^ (BMP2-WT) and BMP2-cKO mice were applied to Alizarin Red staining on day 14. n=3. (C) Western blot analysis of SP7 and OPN protein expression levels in mBMSCs. β-Actin was used as an internal control. n=3. (D-G) qRT-PCR analysis of *Sp7* (D), *Opn* (E), *Runx2* (F) and *Bglap* (G) mRNA expression levels in BMP2-WT and BMP2-cKO cells. *Gapdh* was used as an internal control. n=3, ***P* < 0.01, *****P* < 0.0001.

**Supplementary Fig. 4.** mBMSCs isolated from *Sp7-Cre*; *Bmp2*^fx/fx^ mice decreased the capability of proliferation and adipogenesis.

(A, B) EdU staining assay indicated the higher proportion of proliferating cells in *Bmp2*^fx/fx^ (BMP2-WT) cells. n=3, ***P* < 0.01. (C) CCK-8 assay indicated that the higher cell viability in BMP2-WT cells. n=3, ****P* < 0.001, *****P* < 0.0001. (D-G) qRT-PCR analysis of *Bmp2* (D), *Aiponectin* (E), *Fabp4* (F) and *PPAR-γ* (G) mRNA expression levels in BMP2-WT and *Sp7-Cre*; *Bmp2*^fx/fx^ (BMP2-cKO) cells. *Gapdh* was used as an internal control. n=3, **P* < 0.05, ***P* < 0.01, *****P* < 0.0001.

**Supplementary Fig. 5.** Genomewide profile of BMP2-dependent open chromatin landscape in osteoblast differentiation.

(A, B) Distribution of BMP2-cKO mBMSCs lost (A) and gained (B) NFRs to TSS of nearest genes. (C, D) GO enrichment assay for the nearby genes (within 100 bp) of BMP2-cKO mBMSCs lost (C) and gained (D) NFRs. (E) Top five enriched motifs in deletion of *Bmp2* gained NFRs. PWM, position weighted matrix. TF, transcription factors.

**Supplementary Fig. 6.** Supposed regulatory network of major BMP2-WT enriched transcription factors in osteoblast differentiation.

The BMP2-WT enriched motifs are in hexagon and the relevant transcription factors are in square. The thickness of each edge represents the number of motifs located in the all nearby enhancers to each TF.

**Supplementary Fig. 7.** KLF4-deficient mice exhibited underdeveloped skeletons with abnormal tooth phenotype.

(A) All mice were genotyped by PCR from tail snip DNA. (B-E) The skulls (B), ribs (C), clavicles (D) and mandibles (E) displayed decreased ossification in the *Sp7-Cre*; *Klf4*^fx/fx^ (KLF4-cKO), the clavicles and mandibles of the mutant mice were also underdeveloped. n=3. (F) The forelimbs were shorter but had normal ossification in the KLF4-cKO mice. n=3. (G) Tooth photographic analysis of 16-week-old *Klf4*^fx/fx^ (KLF4-WT) and *Sp7-Cre*; *Klf4*^fx/+^ (KLF4-Het) mice. Black arrowheads indicated incisors were asymmetric with open forked in KLF4-Het mice compared with KLF4-WT. n=6. (H-J) Stereo Microscope analysis (H) and high magnification (I, J) of mandibles from 16-week-old KLF4-WT and KLF4-Het mice. Stereo Microscope analysis indicated defective mandibles and caries in the KLF4-Het mice. n=6.

**Supplementary Fig. 8.** Histology analysis of *Klf4*^fx/fx^ and *Sp7-Cre*; *Klf4*^fx/+^ mice at different ages.

(A-C) H&E staining of mouse femurs from 2-week-old (A), 4-week-old (B) and 16-week-old (C) *Klf4*^fx/fx^ (KLF4-WT) and *Sp7-Cre*; *Klf4*^fx/+^ (KLF4-Het) mice. n=3. (D) Safranin O staining of mouse femurs from 2-week-old, 4-week-old, 8-week-old and 16-week-old KLF4-WT and KLF4-Het mice. n=3.

**Supplementary Fig. 9.** mRNA level of *Klf4* in mBMSCs

qRT-PCR analysis of *Klf4* mRNA expression levels in *Klf4*^fx/fx^ (KLF4-WT) and *Sp7-Cre*; *Klf4*^fx/+^ (KLF4-Het) cells. *Gapdh* was used as an internal control. n=3, *****P* < 0.0001.

**Supplementary Fig. 10.** Genomewide profile of KLF4-dependent open chromatin landscape in osteoblast differentiation.

(A, B) Distribution of KLF4-Het mBMSCs lost (A) and gained (B) NFRs to TSS of nearest genes. (C, D) GO enrichment assay for the nearby genes (within 100 bp) of KLF4-Het mBMSCs lost (C) and gained (D) NFRs. (E) Top six enriched motifs in deletion of *Klf4* gained NFRs. PWM, position weighted matrix. TF, transcription factors.

**Supplementary Fig. 11.** Supposed regulatory network of major KLF4-WT enriched transcription factors in osteoblast differentiation.

The KLF4-WT enriched motifs are in hexagon and the relevant transcription factors are in square. The thickness of each edge represents the number of motifs located in the all nearby enhancers to each TF.

**Supplementary Fig. 12.** *Klf4* is a BMP2-dependent transcription factor.

(A) Venn diagram showing the lost NFRs overlap of *Sp7-Cre*; *Bmp2*^fx/fx^ (BMP2-cKO) and *Sp7-Cre*; *Klf4*^fx/+^ (KLF4-Het) in mBMSCs. (B) GO enrichment assay for the nearby genes (within 100 bp) of BMP2-cKO lost NFRs intersect with KLF4-Het lost NFRs. (C) Top five enriched motifs in BMP2-cKO lost NFRs intersect with KLF4-Het lost NFRs. PWM, position weighted matrix. TF, transcription factors.

**Supplementary Fig. 13.** KLF4 interacts with coactivators to initiate osteoblast differentiation.

(A) Dot plot showing the GO enrichment assay for the nearby genes of KLF occupied with DLX motif in *Klf4* deficiency lost NFRs. (B) Dot plot showing the GO enrichment assay for the nearby genes of KLF occupied with MADS motif in *Klf4* deficiency lost NFRs. (C) Dot plot showing the GO enrichment assay for the nearby genes of KLF occupied with CEBPE motif in KLF4 deficiency lost NFRs. (D) MC3T3-E1 cells were immunoprecipitated with ant-RUNX2 and anti-KLF4, and immunoblotted with the indicated antibodies. n=3.
