# Supplementary figures and images for: BMP2-dependent gene regulatory network analysis reveals Klf4 as a novel transcription factor of osteoblast differentiation
Source: Cell Death Dis. 2021 Feb 19;12(2):197. doi: 10.1038/s41419-021-03480-7 (PMC7895980; doi:10.1038/s41419-021-03480-7)

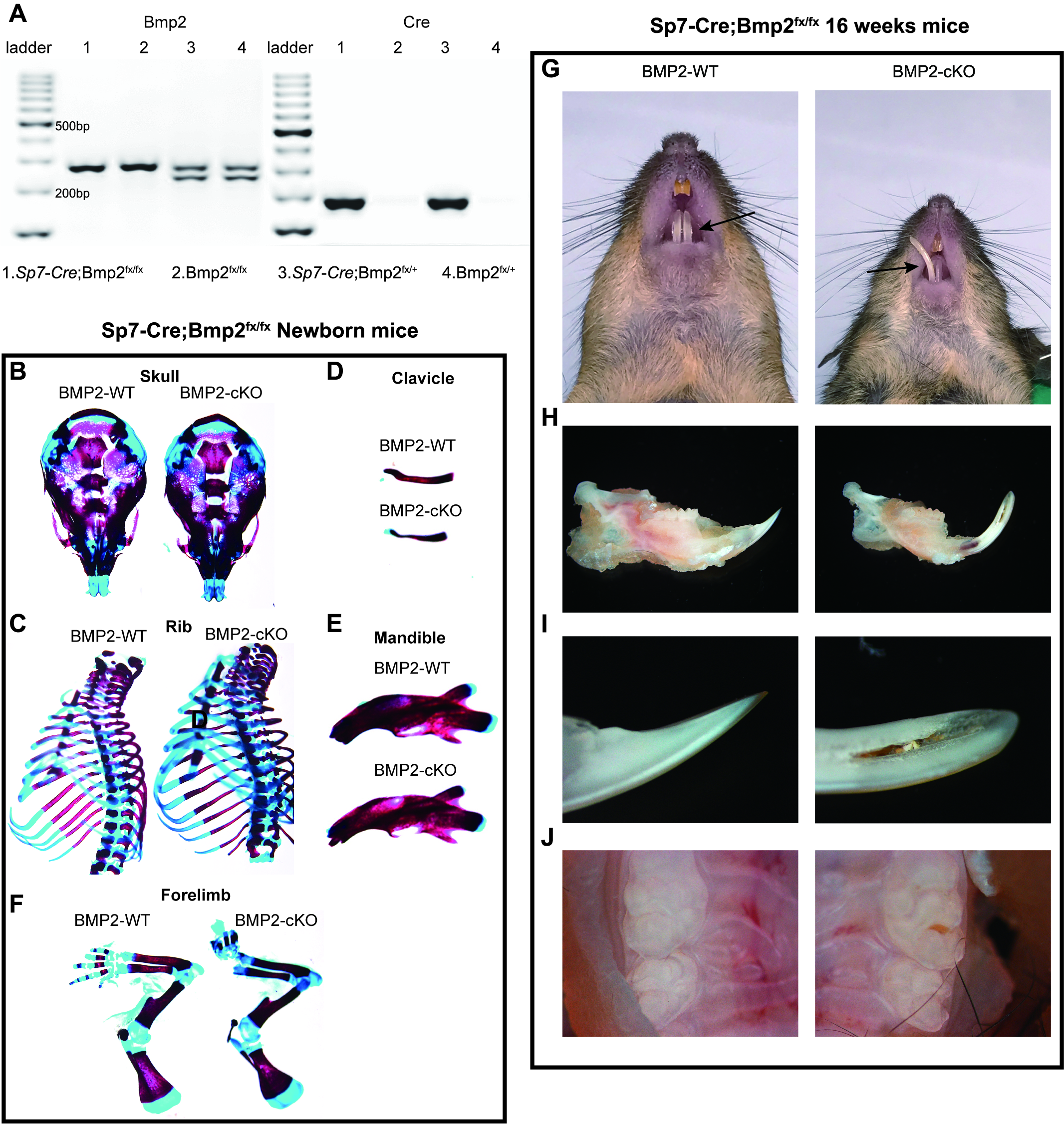

Supplement: Supplementary file 2 — Suppl figure 1 [file 41419_2021_3480_MOESM2_ESM.tif]

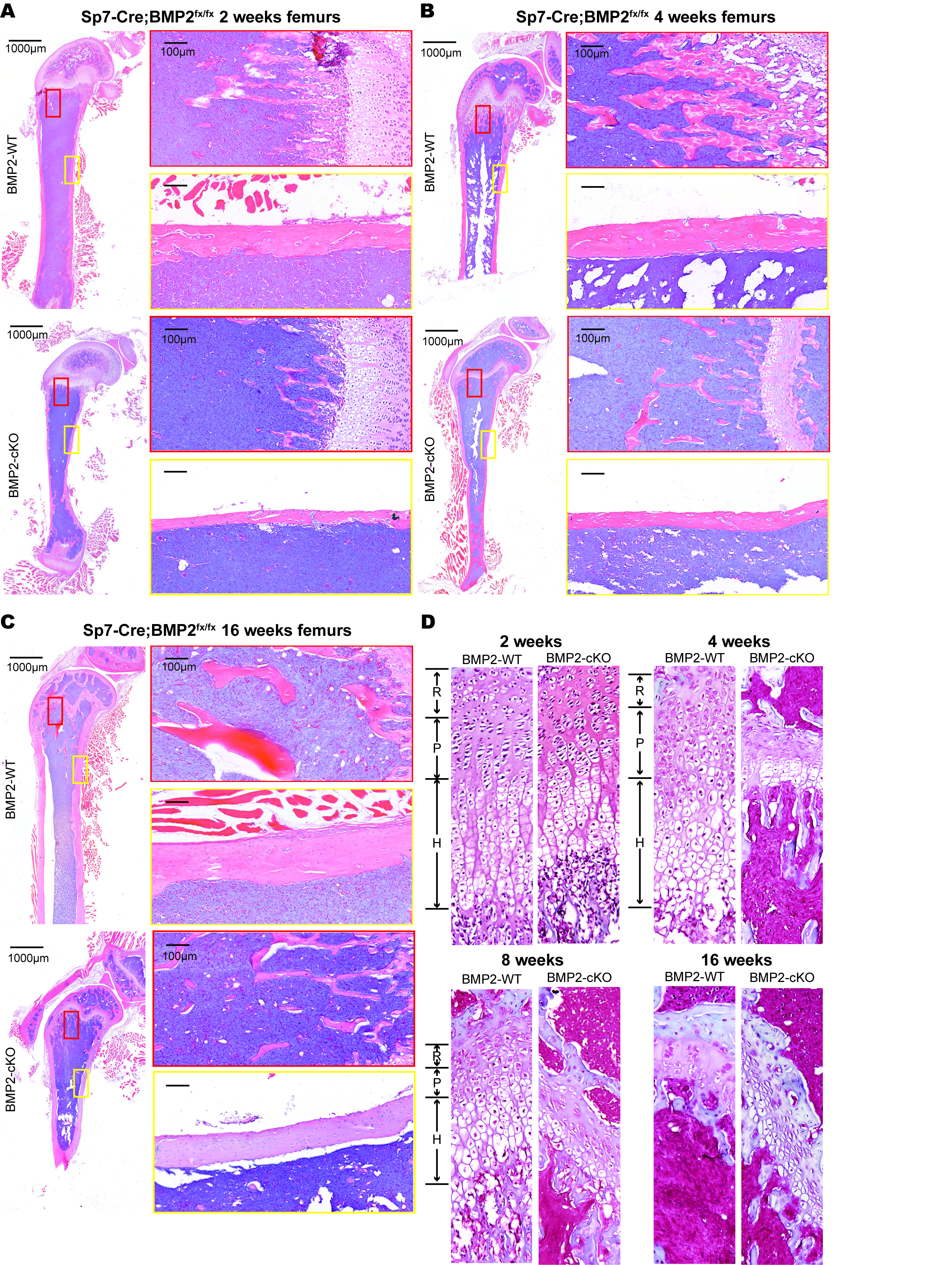

Supplement: Supplementary file 3 — Suppl figure 2 [file 41419_2021_3480_MOESM3_ESM.tif]

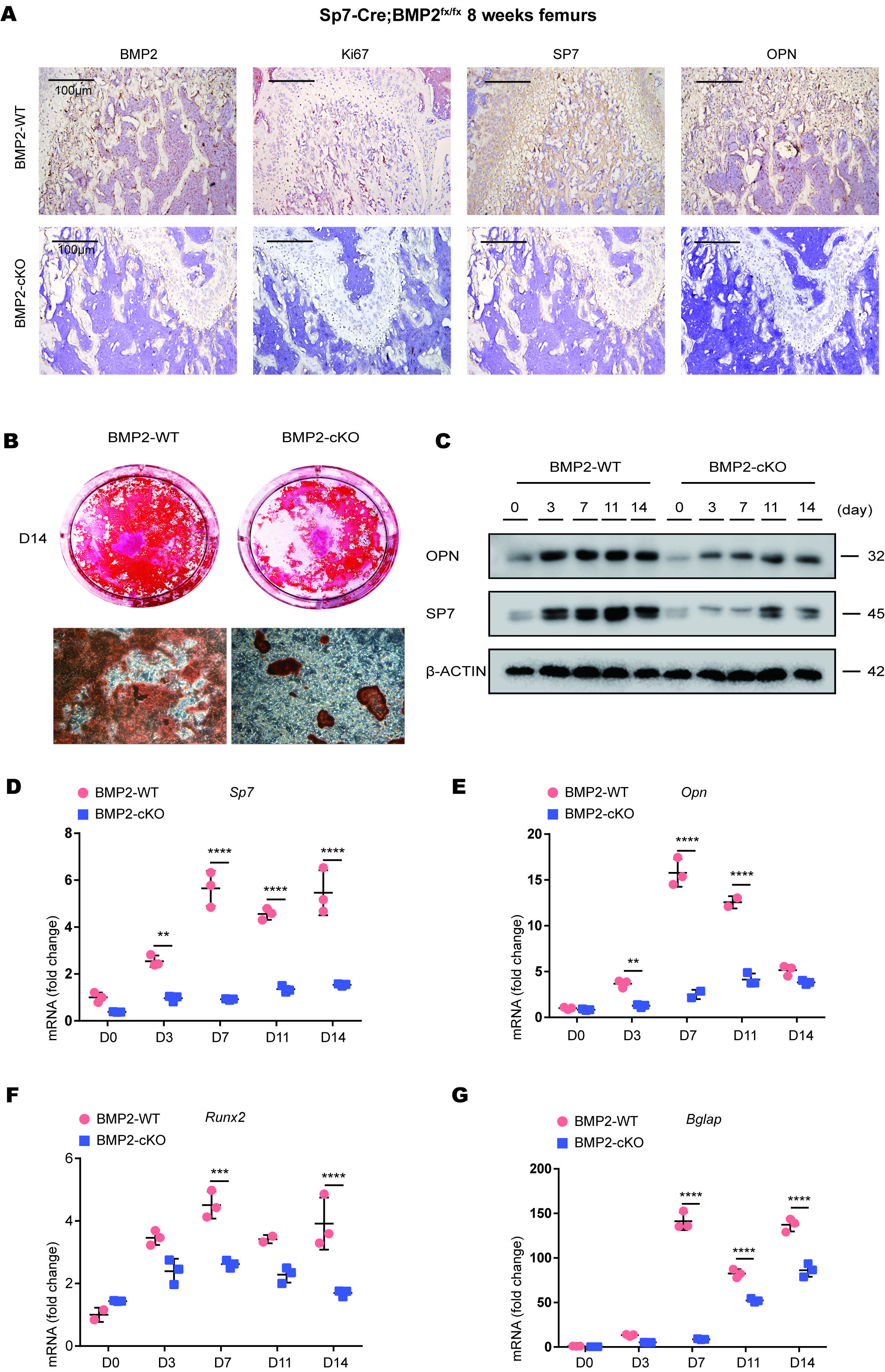

Supplement: Supplementary file 4 — Suppl figure 3 [file 41419_2021_3480_MOESM4_ESM.tif]

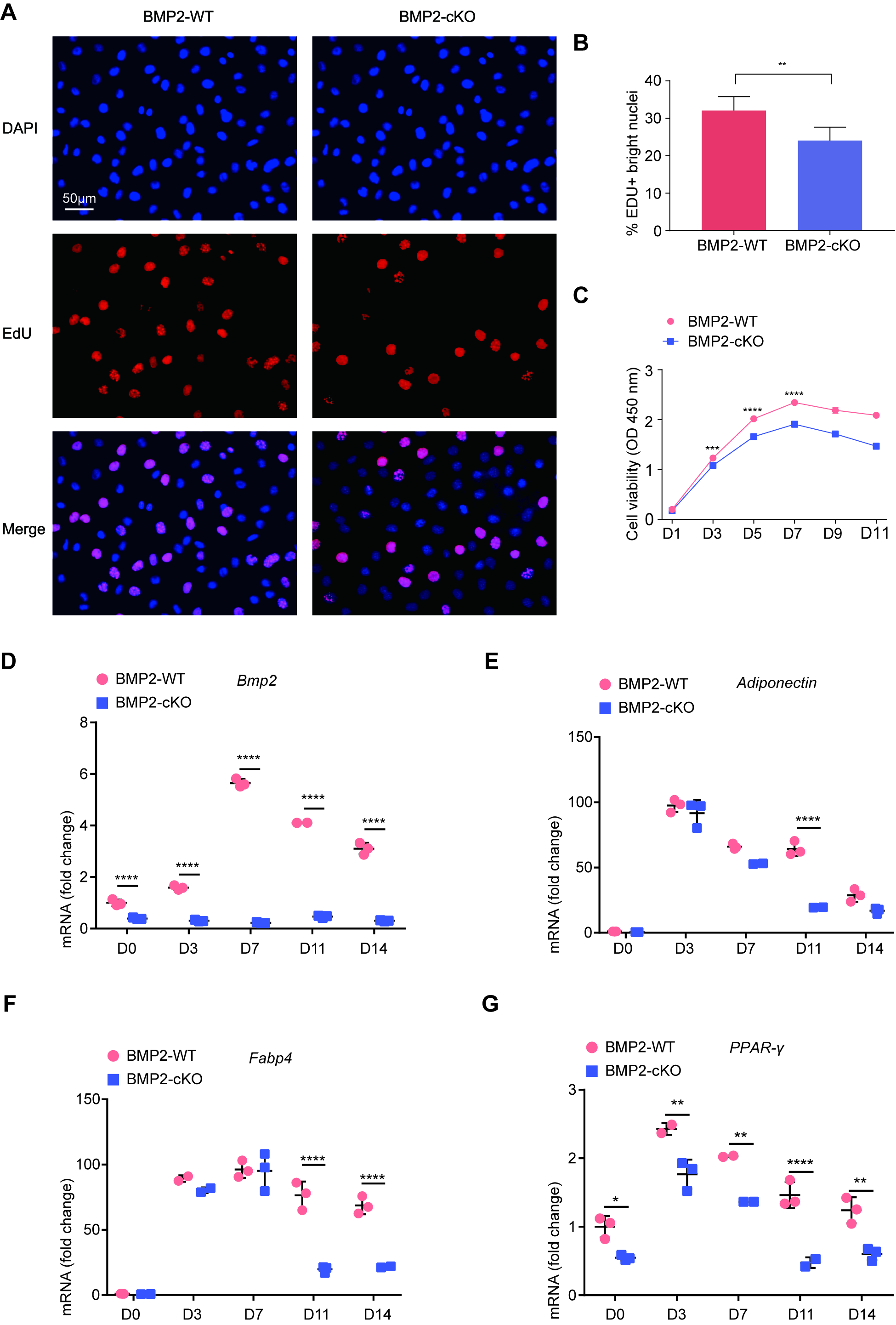

Supplement: Supplementary file 5 — Suppl figure 4 [file 41419_2021_3480_MOESM5_ESM.tif]

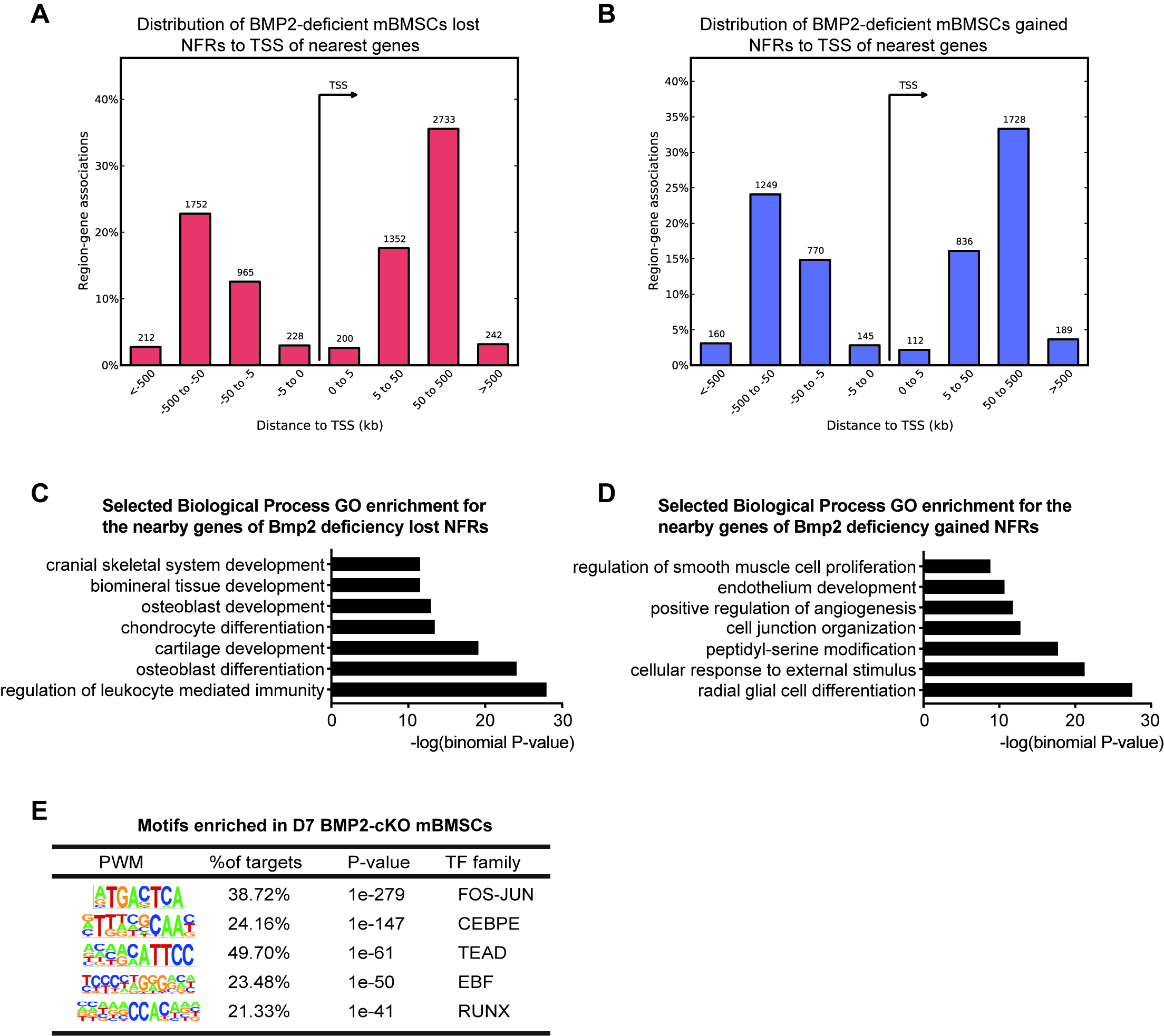

Supplement: Supplementary file 6 — Suppl figure 5 [file 41419_2021_3480_MOESM6_ESM.tif]

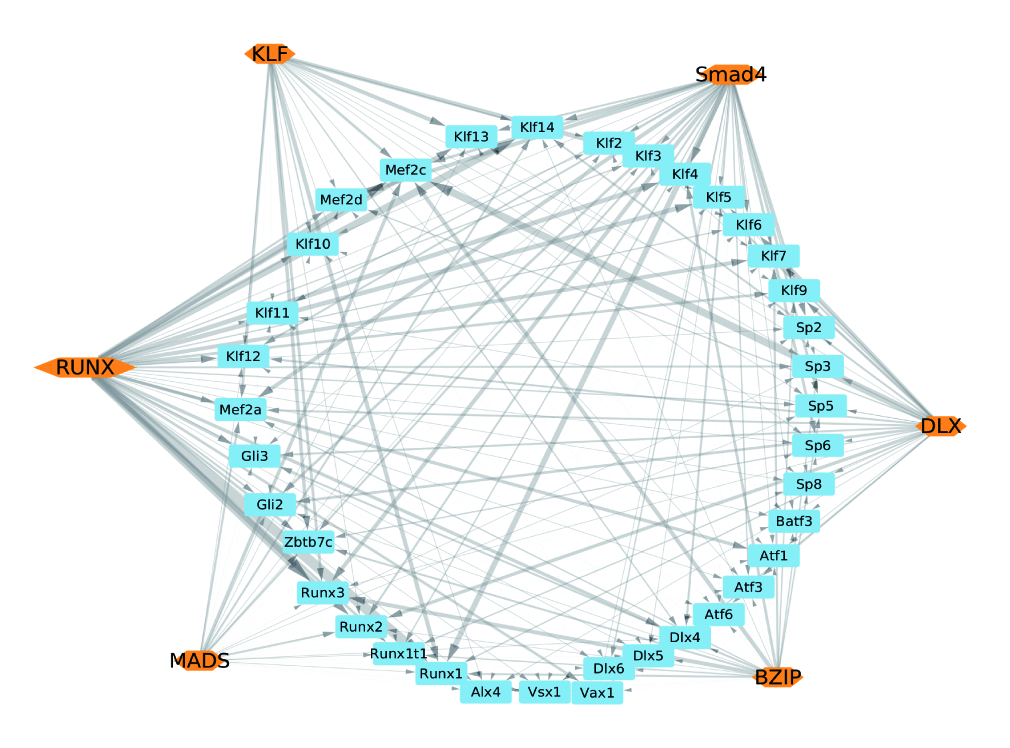

Supplement: Supplementary file 7 — Suppl figure 6 [file 41419_2021_3480_MOESM7_ESM.tif]

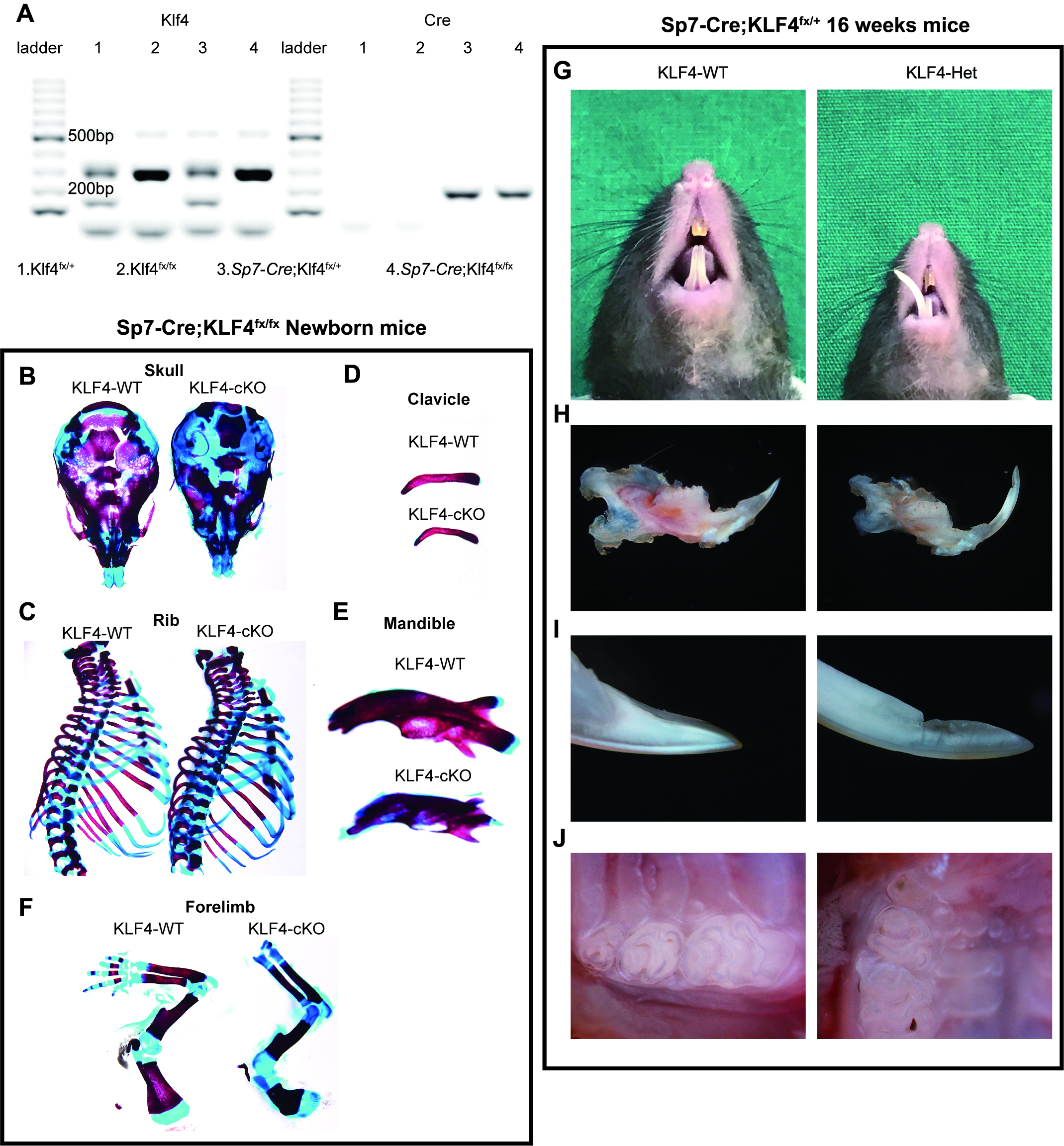

Supplement: Supplementary file 8 — Suppl figure 7 [file 41419_2021_3480_MOESM8_ESM.tif]

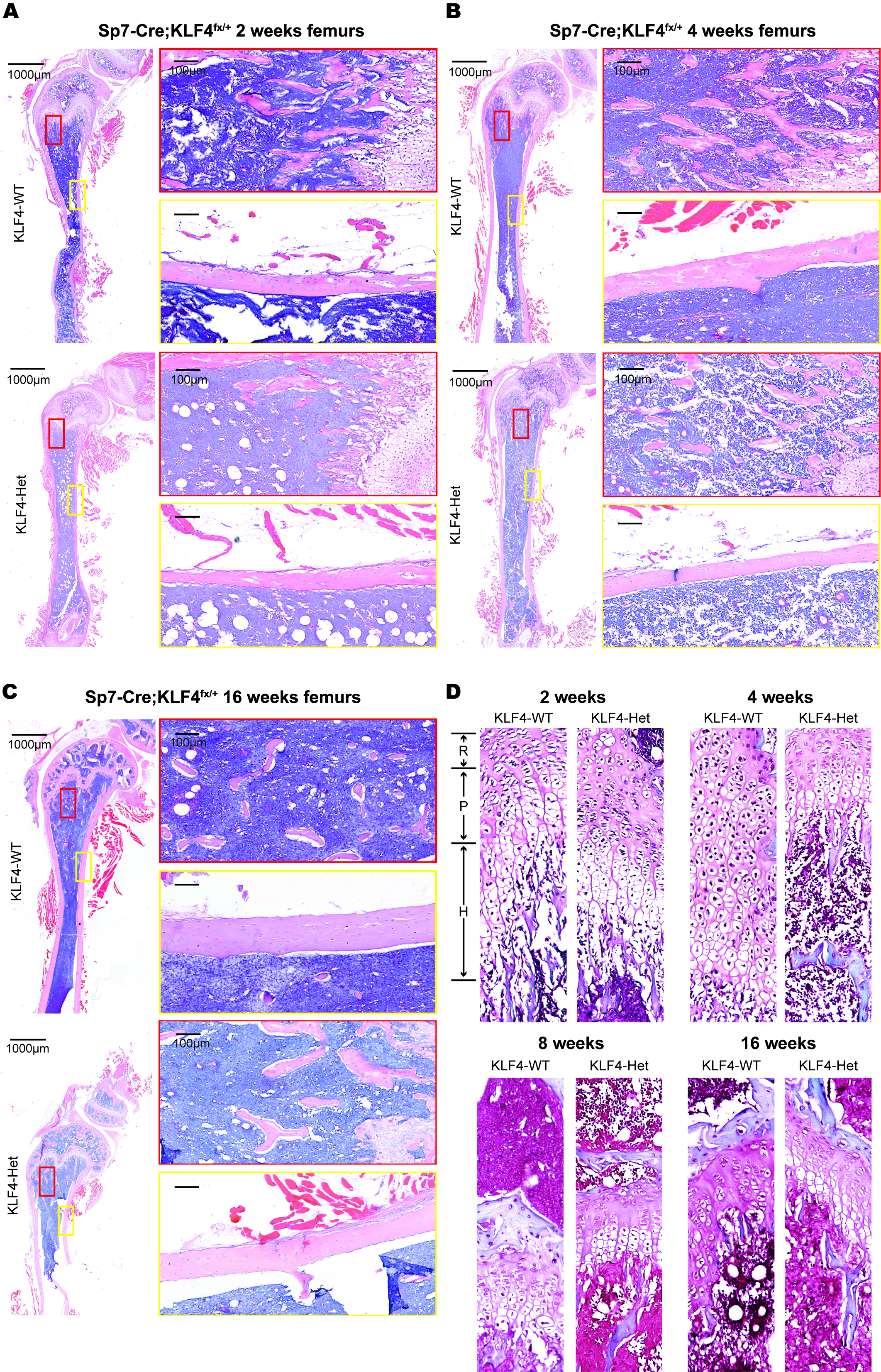

Supplement: Supplementary file 9 — Suppl figure 8 [file 41419_2021_3480_MOESM9_ESM.tif]

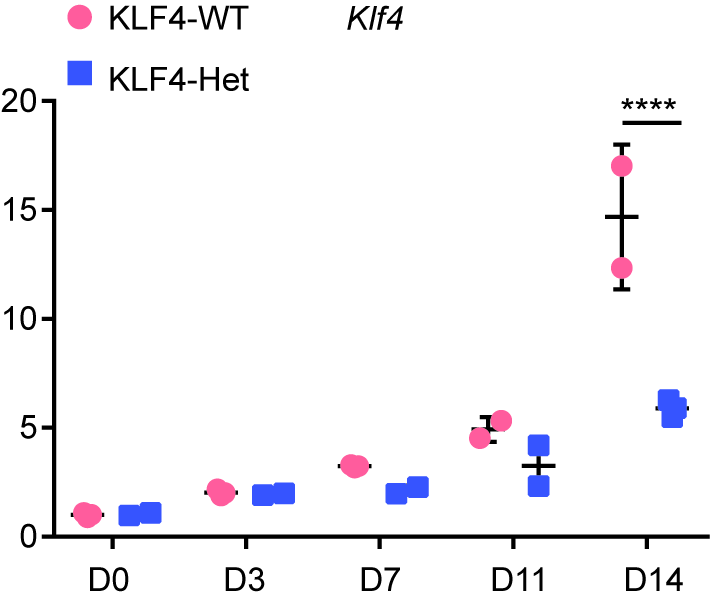

Supplement: Supplementary file 10 — Suppl figure 9 [file 41419_2021_3480_MOESM10_ESM.tif]

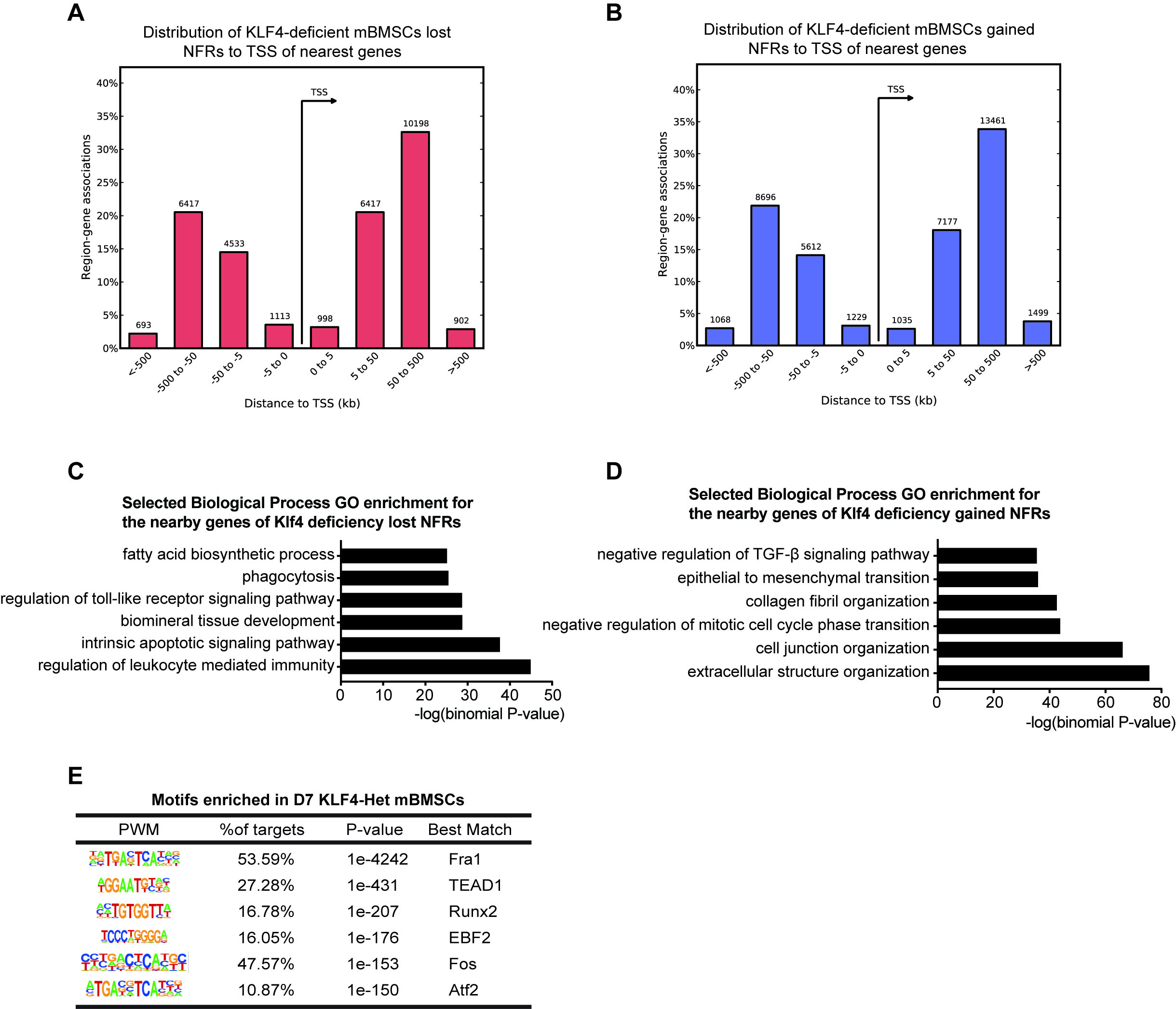

Supplement: Supplementary file 11 — Suppl figure 10 [file 41419_2021_3480_MOESM11_ESM.tif]

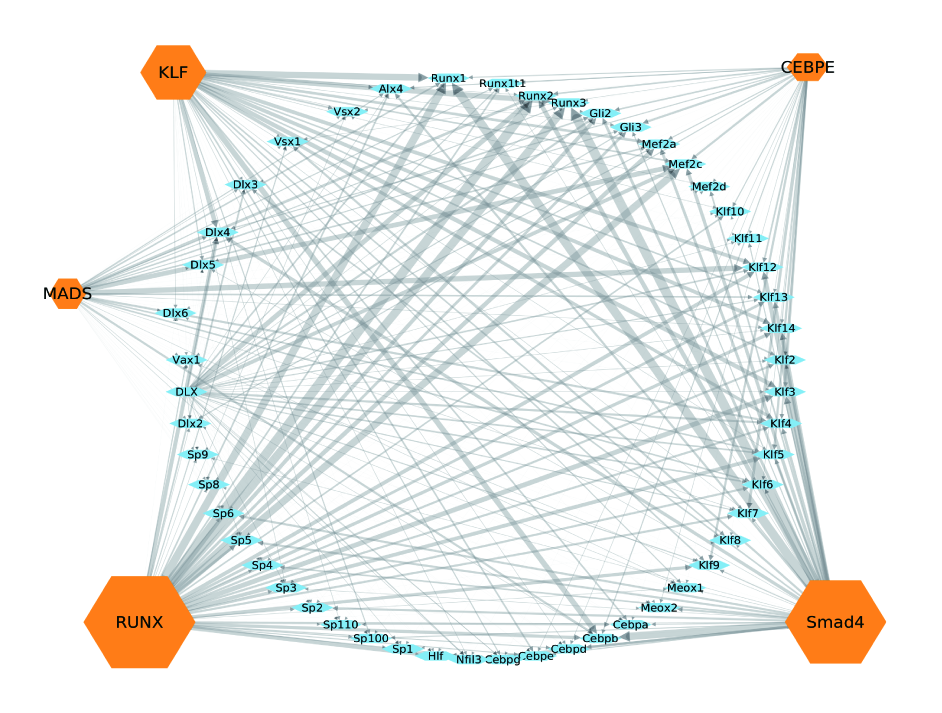

Supplement: Supplementary file 12 — Suppl figure 11 [file 41419_2021_3480_MOESM12_ESM.tif]

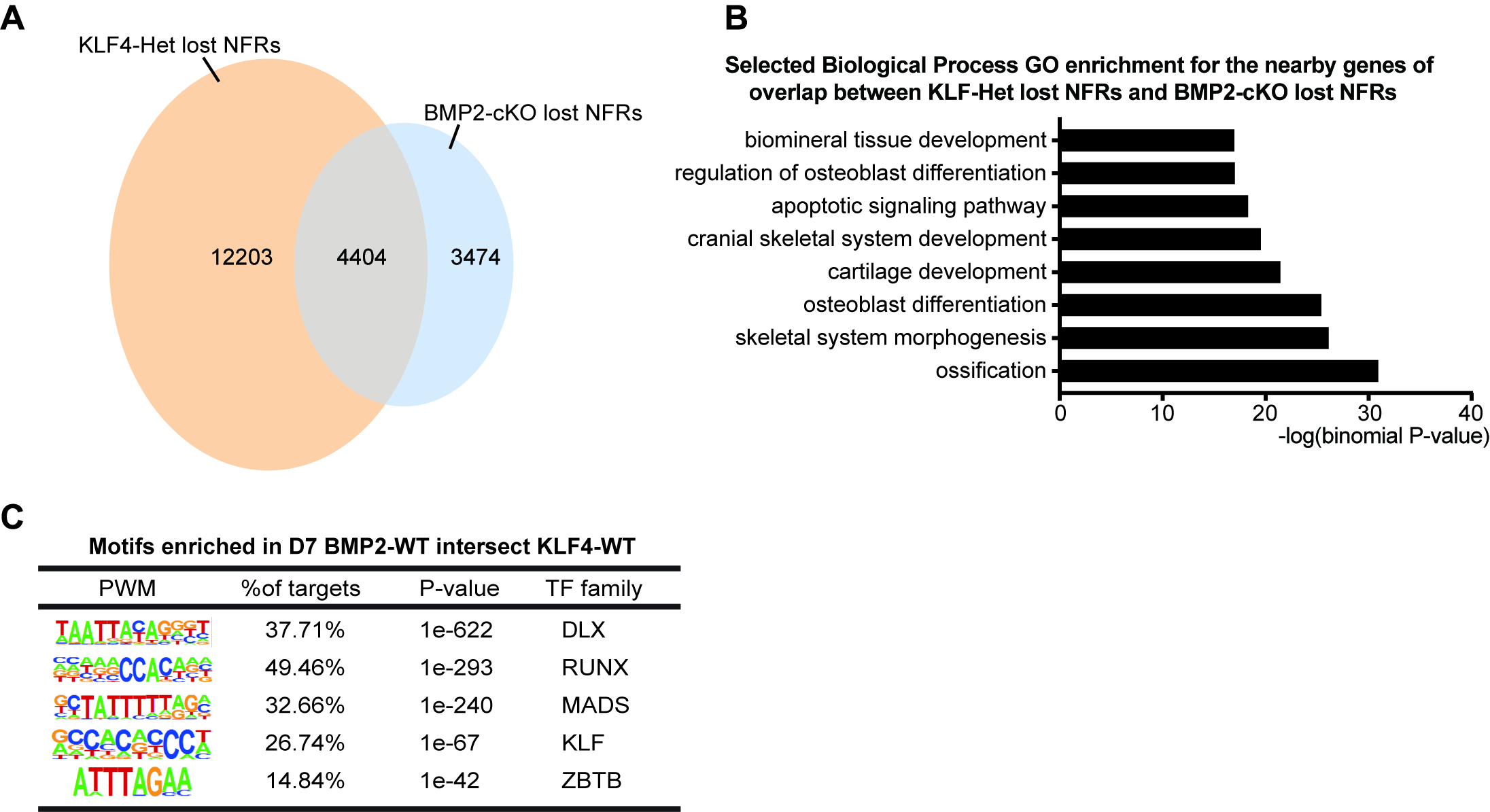

Supplement: Supplementary file 13 — Suppl figure 12 [file 41419_2021_3480_MOESM13_ESM.tif]

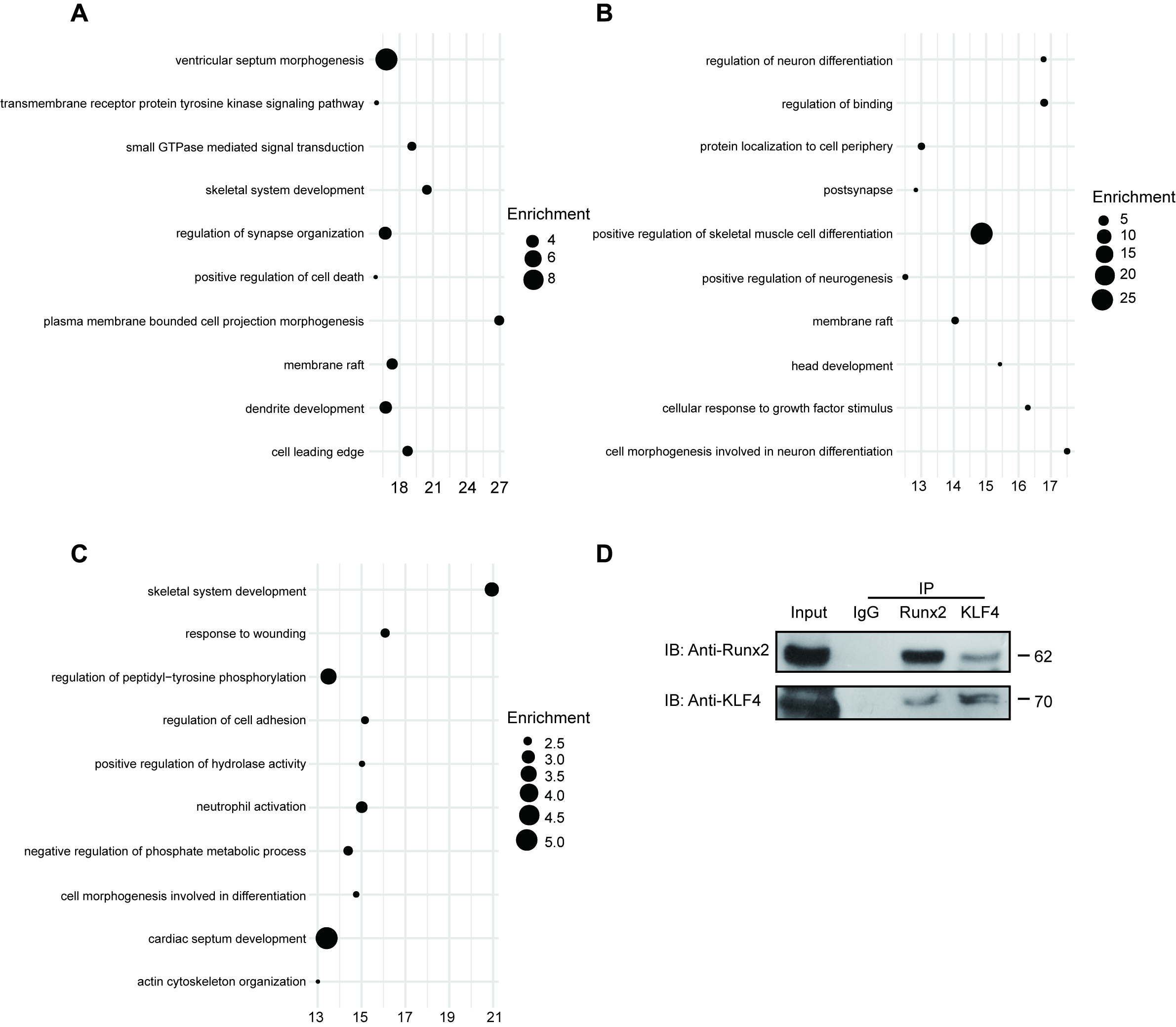

Supplement: Supplementary file 14 — Suppl figure 13 [file 41419_2021_3480_MOESM14_ESM.tif]
